# Hallmarks of liver cancer: Therapeutic implications

**DOI:** 10.1016/j.cell.2026.03.001

**Published:** 2026-04-16

**Authors:** Josep M. Llovet, Roser Pinyol, Silvia Affo, Mark Yarchoan, Gregory J. Gores, Robin Kate Kelley, Scott W. Lowe, Daniela Sia, Augusto Villanueva

**Affiliations:** 1 Mount Sinai Liver Cancer Program, Divisions of Liver Diseases, Department of Medicine, Tisch Cancer Institute, Icahn School of Medicine at Mount Sinai, New York, NY, USA; 2 Liver Cancer Translational Research Group, Liver Unit, Institut d’Investigacions Biomèdiques August Pi i Sunyer (IDIBAPS), Hospital Clínic, Universitat de Barcelona, Barcelona, Catalonia, Spain; 3 Institució Catalana de Recerca i Estudis Avançats, Barcelona, Catalonia, Spain; 4 Tumor Microenvironment Plasticity and Heterogeneity Research Group, Institut d’Investigacions Biomediques August Pi i Sunyer (IDIBAPS), Barcelona, Spain; 5 Department of Medical Oncology, Sidney Kimmel Comprehensive Cancer Center, Johns Hopkins University, Baltimore, MD, USA; 6 Division of Gastroenterology and Hepatology, Mayo Clinic, Rochester, MN, USA; 7 Helen Diller Family Comprehensive Cancer Center, University of California, San Francisco, San Francisco, CA, USA; 8 Cancer Biology and Genetics Program, Sloan Kettering Institute, Memorial Sloan Kettering Cancer Center, New York, NY 10065, USA; 9 Division of Gastroenterology and Hepatology, Perlmutter Cancer Center, Department of Medicine, New York University Grossman School of Medicine, New York, NY, USA; 10 Howard Hughes Medical Institute, Memorial Sloan Kettering Cancer Center, New York, NY 10065, USA

## Abstract

The hallmarks of cancer, first proposed in 2000, have since provided a unified framework for understanding the complexity of carcinogenesis. This conceptual model has profoundly influenced the treatment landscape of primary liver cancer, which includes hepatocellular carcinoma (HCC, ~85%) and intrahepatic cholangiocarcinoma (iCCA, 10%)—malignancies with high mortality. Key hallmarks exhibited by HCC include sustaining proliferative signaling, inducing or accessing vasculature, and avoiding immune detection. Over the past two decades, outcomes for patients with advanced HCC have significantly improved with immunotherapies. iCCA is characterized by hallmarks such as sustaining proliferative signaling, deregulating cellular metabolism, and avoiding immune detection. Unlike HCC, roughly 45% of iCCA harbor alterations amenable to precision oncology approaches, including fibroblast growth factor receptor 2 (FGFR2) fusions, isocitrate dehydrogenase 1 (*IDH1*) mutations, ERBB2 alterations, and BRAF mutations. In this review, we explore how this framework has reshaped liver cancer care and discuss the resulting breakthroughs in management and emerging directions that may further improve therapeutic strategies.

## OVERVIEW OF HALLMARKS AND THERAPIES IN LIVER CANCER

With nearly 1 million new cases annually worldwide, liver cancer is the third leading cause of death, and its incidence is increasing globally.^1,2^ Assuming current rates persist, new annual cases are expected to reach 1.5 million by 2050.^1^ The most common types of primary liver cancer are hepatocellular carcinoma (HCC, 85%–90%) and intrahepatic cholangiocarcinoma (iCCA, 10%–15%). Both may arise in the context of chronic liver inflammation; however, HCC generally develops in cirrhotic livers due to hepatitis B virus (HBV) or hepatitis C virus (HCV) infection, alcohol-related liver disease (ALD), and metabolic dysfunction-associated steatotic liver disease (MASLD),^3^ whereas in iCCA, identifiable risk factors are only found in 30%–40% of cases. HCC and iCCA profoundly differ in their molecular pathogenesis and, consequently, in their treatment options. Both tumors exhibit a low mutational burden (less than four mutations per megabase [mut/MB] of the coding genome) and an immunosuppressive tumor microenvironment (TME), usually refractory to single-agent immunotherapies. Targetable genomic drivers have been identified in 45% of iCCA cases, marking a major success in precision oncology for tumors harboring activating alterations in BRAF, fibroblast growth factor receptor (*FGFR*), *ERBB2*, and isocitrate dehydrogenase 1 (*IDH1*).^4^ Conversely, despite ~25% of HCCs containing at least one potentially actionable mutation,^5^ no targeted therapy has been approved thus far.

Key cancer hallmarks associated with HCC include sustained proliferative signaling, induction of vasculature access, and avoidance of immune destruction. Targeting these hallmarks selectively has led to clinical improvements in patient survival. For iCCA, a more refined precision medicine has emerged through the identification of recurrent, druggable alterations affecting cancer hallmarks. This review examines the cancer hallmarks^6–8^ of the two main liver cancer types, HCC and iCCA, and how they have shaped patient classification, current treatment paradigms, and guided the development of novel treatment strategies. In addition, we integrate these hallmarks with therapeutic advances that have yielded clinical benefits.

### HCC

HCC is a global health problem, with an incidence of ~8 cases per 100,000 inhabitants.^9^ HCC arises in the setting of chronic liver injury, typically after decades of inflammation, cell death, and fibrosis. The major historical risk factors are chronic HBV and HCV infection, alcohol use, and hepatotoxins such as aflatoxin B1.^3^ More recently, metabolic dysfunction-associated steatohepatitis (MASH, formerly NASH), linked to obesity and diabetes, has emerged as a rapidly growing cause, particularly in Western countries.^10,11^

Prevention of HCC development can be achieved through universal HBV vaccination and antiviral therapies for patients with chronic HBV or HCV infections.^3^ Biannual surveillance with ultrasound and α-fetoprotein (AFP) blood testing is recommended for high-risk patients, particularly those with liver cirrhosis, as it has been associated with early tumor detection and increased applicability of curative therapies.^3^ However, surveillance program implementation reaches fewer than 50% of the target population.^3^ Blood-based biomarkers, such as DNA methylation markers or ctDNA detection, are not yet ready for clinical practice.

Overall, only 30% of HCC patients can be cured, and the 5-year survival rate remains below 30%. Advances in surgical techniques and locoregional therapies have improved outcomes for early- and intermediate-stage tumors, with median overall survival (mOS) reaching >60 and ~30 months, respectively.^3^ Nonetheless, most patients are diagnosed at or progress to advanced-stage disease, which has a poor prognosis (mOS: ~8 months).^3^ Systemic therapies have revolutionized the management of advanced HCC over the past 25 years. Sorafenib, a multi-tyrosine kinase inhibitor targeting BRAF, vascular endothelial growth factor 2 or 3 (VEGFR2/3), and platelet-derived growth factor receptor B (PDGFRB), was the first systemic therapy to improve survival in advanced stages, and together with lenvatinib, another multi-tyrosine kinase inhibitor targeting VEGFR1/2/3, FGFR1/2/3/4, and PDGFR enhanced first-line outcomes (mOS: 13–19 months).^3^ Subsequently, regorafenib, cabozantinib, and ramucirumab improved outcomes in patients progressing after sorafenib (mOS: 9–11 months). More recently, immunotherapies have transformed the management of advanced disease, with combinations of checkpoint inhibitors (ICIs) and VEGF-A inhibitors (atezolizumab plus bevacizumab) or dual checkpoint inhibitor regimens (durvalumab plus tremelimumab or nivolumab plus ipilimumab) further extending survival (mOS: ~16–23 months), achieving a 4-year survival tail of ~25% and improving quality of life.^3,12^ We next discuss the intricate interplay between the cancer hallmarks,^6–8^ HCC, and therapeutic interventions.

### Hallmarks: Sustained proliferative signaling, genome instability and mutation, and metabolism deregulation

#### Hepatocarcinogenesis

The majority of HCCs arise in the context of cirrhosis^3^ and evolve through a sequence of well-defined histopathological stages, beginning with dysplastic nodules that can ultimately progress to malignant tumors.^13,14^ Genetic and epigenetic alterations occur primarily in hepatocytes, although liver stem cells have also been directly implicated.^3,15,16^ Through a process of necrosis and regeneration, oxidative stress, and blockage of apoptosis, the hepatocytes acquire molecular aberrations, especially in the context of an immunosuppressive tissue environment and fibrosis.^17^ Telomerase reverse transcriptase (*TERT*)-activating mutations, chromosomal gains (1q, 8q) and losses (8p, 22p), and epigenetic changes (APC, SOCS1, TSPYL5) are reported gatekeepers in 30%–50% of dysplastic nodules.^3,13^ In addition, germline variants associated with advanced liver disease—including PNPLA3, TM6SF2, and HSD17B—confer increased HCC risk, while environmental carcinogens such as aflatoxin B1 and aristolochic acid are genotoxic compounds inducing characteristic mutations.^3^ Experimental studies indicate that hepatocyte spatial organization within the three functional zones of the liver—defined by proximity to the blood supply and associated metabolic programs—modulates cancer risk, with zone 3 hepatocytes exhibiting increased susceptibility to tumor initiation, particularly driven by *CTNNB1* mutations, which is a gene that encodes β-catenin and is a critical effector of the Wnt pathway.^18,19^ Upon accumulation of molecular aberrations, the onset of HCC occurs, with very few actionable truncal mutations per tumor (Table 1).

#### Key molecular alterations and subclasses

**Figure 1.**
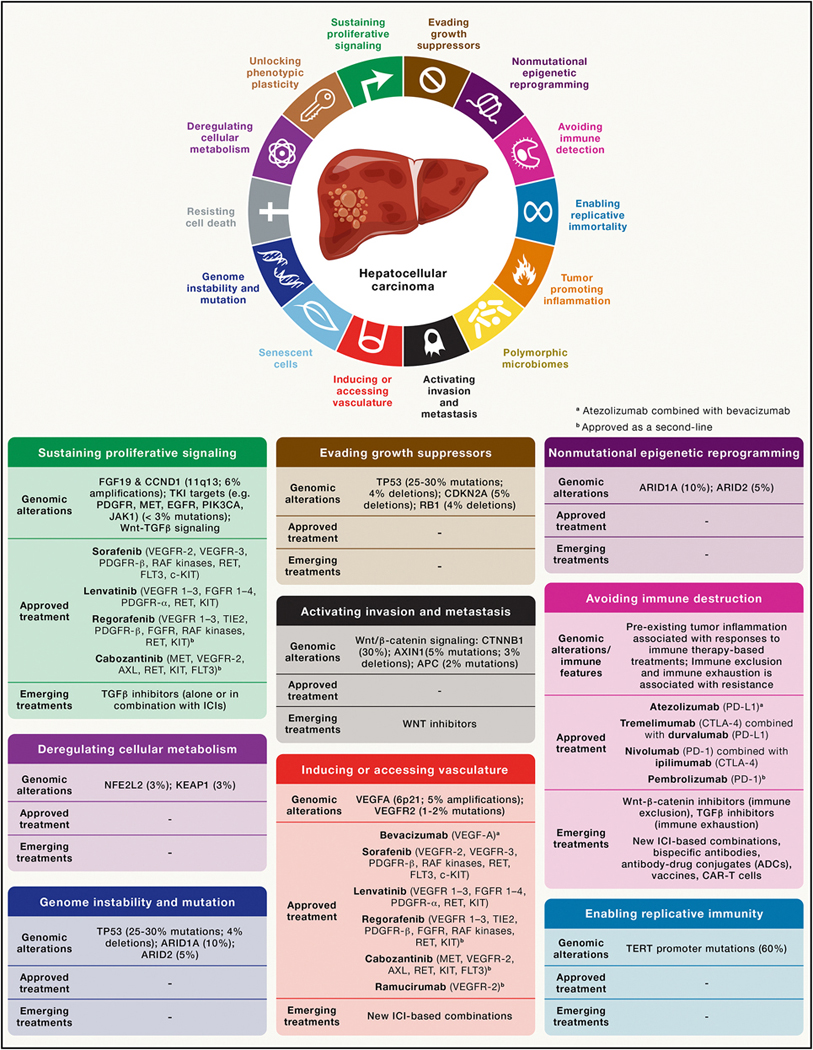
Integrated overview of cancer hallmarks, genomic alterations, and approved and emerging therapies in HCC Major cancer hallmarks acquired during HCC development, highlighting key genomic alterations and dysregulated pathways associated with each hallmark. Approved systemic therapies and emerging treatment strategies are mapped to the corresponding biological processes.

The genetic landscape of HCC is defined by a low mutation burden, with recurrent lesions in key signaling pathways.^3,13,14,21^ Tumors typically harbor 60–70 coding alterations, but only a minority act as *bona fide* drivers. Prevalent genomic alterations in HCC are summarized in Table 1. The most frequent mutations in HCC occur in the promoter region of the *TERT* gene (~60%).^22,23^ These mutations reactivate the telomerase and thereby enable the cancer hallmark of replicative immortality^6–8^ (Figure 1). Telomerase reactivation can also occur through alternative mechanisms. In this context, chronic HBV infection contributes not only to cirrhosis but also to direct oncogenic effects, as the HBV virus can integrate into the human genome, frequently within the *TERT* promoter (25% of cases), activating the telomerase and promoting malignant transformation.^24^ Inactivation of *TP53* (~25%–30%) disables anti-proliferative programs, enables oncogenic plasticity, and promotes genomic instability and aggressive growth.^25^ Mutations in chromatin remodelers such as *ARID1A* and *ARID2* (10% and 5%) disrupt enhancer regulation and alter the tumor environment,^26^ thereby contributing to two emerging hallmarks: non-mutational epigenetic reprogramming, through widespread remodeling of chromatin accessibility; and genomic instability, by weakening DNA-damage surveillance and replication fidelity (Figure 1).^6–8^ By contrast, even though *RAS* mutations are uncommon (<5%), RAS-mitogen-activated protein kinase (MAPK) signaling can be activated through receptor tyrosine kinases or copy-number gains,^5,27^ contributing to the sustained proliferative signaling hallmark. Mutations in canonical therapeutic targets, such as *MET*, epidermal growth factor receptor (*EGFR*), or *PIK3CA*, are also rare (<3%),^3^ yet when present, they similarly drive proliferative as well as vascularization programs. To date, none of these recurrent alterations are actionable with selectively approved therapies (as opposed to multikinase inhibitors), underscoring why HCC has yet to benefit broadly from precision oncology.

Copy-number alterations (CNAs) shape HCC by changing chromosomal dosage impacting multiple genes simultaneously and is associated with the hallmark of genome instability (Figure 1). Focal CNAs are more strongly associated with proliferation markers, while broad CNAs are linked to immune evasion and reduced responses to immunotherapy.^28,29^ High-level focal amplifications have been reported for HCC being the most prevalent in 6p21 (6%) and 11q13 (6%).^5,27^ Functional studies demonstrate that most CNAs affect more than a single driver. For example, 11q13 amplifications encompass *CCND1* and *FGF19*, while 11q22 amplifications target both *YAP* and *cIAP*.^5,27,30,31^ Recurrent broad chromosomal gains occur at 1q, 8q, 11q13, and 11q22, whereas frequent deletions involve 8p22 and 17p13.^14,29^ Recurrent 8p deletions eliminate a cluster of haploinsufficient tumor suppressors including DLC1,^32^ and 17p loss disables *TP53* together with additional linked genes,^33^ disrupting DNA-damage responses and apoptotic surveillance and thereby contributing to the genome instability and mutation hallmark as well as evading growth suppressors hallmark (Figure 1). Furthermore, CNAs are strongly associated with *TP53* mutations in HCC and other cancers,^29^ and many involve established drivers such as *MYC* and *TP53* loss, alterations that cooperate to accelerate hepatocarcinogenesis in mice.^34^ Collectively, these findings highlight that CNAs in HCC rarely act through single loci but instead remodel oncogenic programs by amplifying or deleting clusters of functionally cooperative genes involved in cell proliferation.

Epigenetic deregulation provides another critical dimension to hepatocarcinogenesis. *CDKN2A* mutations and promoter hypermethylation of CDKN2A/p16INK4A silence a key cell-cycle checkpoint regulator,^35^ consistent with the evading growth suppressor hallmark (Figure 1). In contrast, global DNA hypomethylation disrupts chromatin integrity and promotes chromosomal instability, defining features of the genome instability and mutation hallmark, and correlates with poor prognosis.^29,36,37^

Broad chromatin alterations are frequently observed in HCC,^3,27,29^ consistent with the notion that shifts in cell state are tightly coupled to disease origins. In some cases, genetic mutations act in concert with, or directly promote, epigenetic reprogramming. For example, *TP53* inactivation in murine hepatocytes permits epigenetic plasticity, driving distinct liver cancer phenotypes depending on the initiating oncogenic insult.^38^ Recurrent alterations in histone-modifying enzymes further reinforce malignant transcriptional programs. *EZH2* overexpression or *KDM6A* inactivation disrupts regulation of repressive *H3K27* trimethylation and silences tumor suppressor networks^39^; *SETDB1* amplification enforces repressive chromatin states; and loss of *ARID1A* perturbs nucleosome remodeling.^35^ Collectively, these changes not only sustain proliferative transcriptional outputs but also create subtype-specific dependencies that may be therapeutically exploitable.^40^

**Figure 2.**
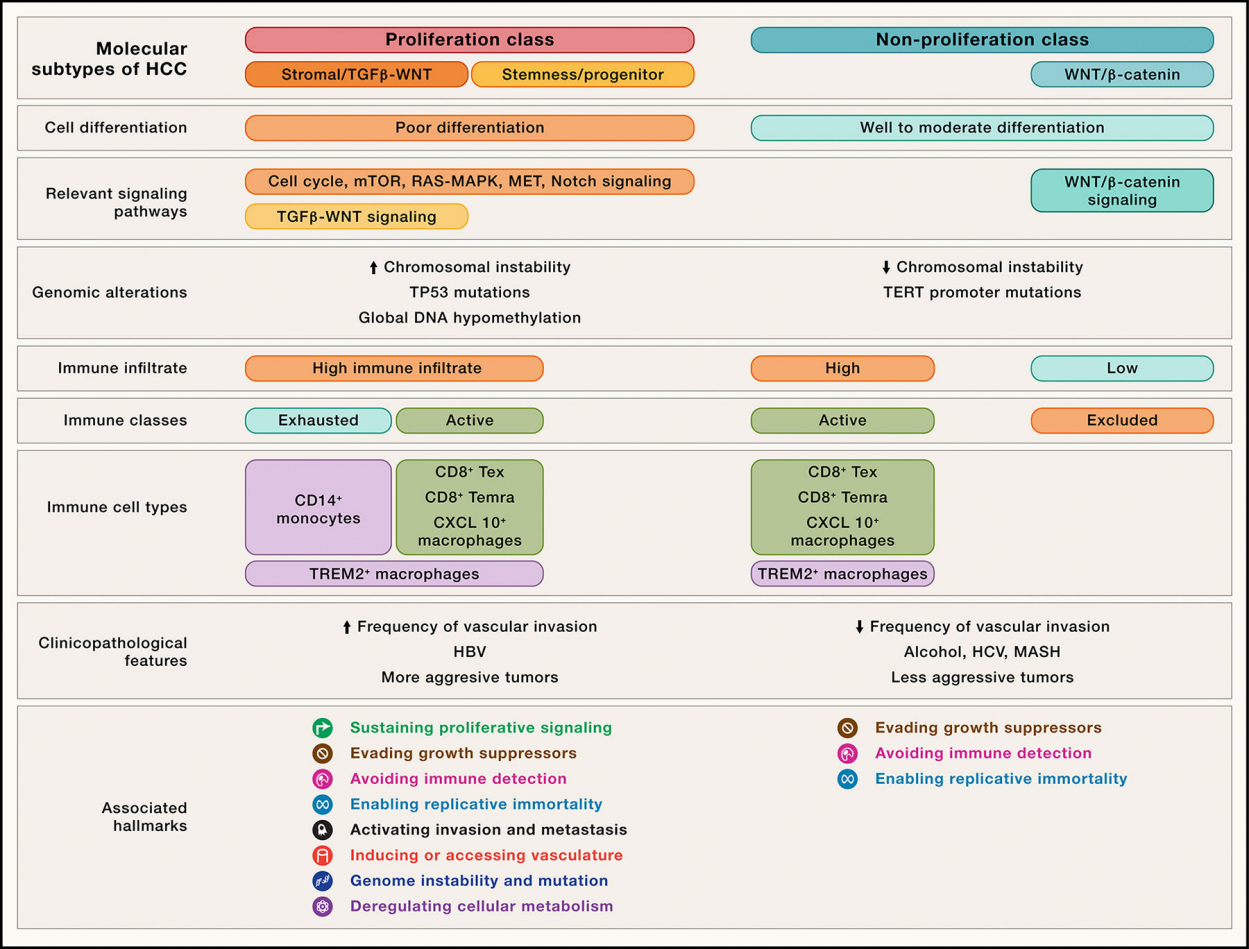
HCC molecular subclasses and their associated hallmarks, mutations, and immune profiles HCC can be divided into two main molecular subclasses based on transcriptomic phenotypes: proliferation and non-proliferation. The proliferation class includes more aggressive tumors, with poor histological differentiation, high vascular invasion, and elevated AFP levels. These tumors often exhibit either activation of the non-canonical Wnt-TGF-β pathway, associated with an immune-exhausted phenotype, or a progenitor-like profile. In contrast, the non-proliferation class is characterized by less aggressive tumors with better differentiation, lower AFP levels, and reduced vascular invasion. This group frequently harbors *CTNNB1* mutations and Wnt-β-catenin pathway activation, which drive an immune-excluded phenotype with low immune cell infiltration. Immune-active tumors are typically enriched in terminally differentiated effector memory T cells re-expressing RA (TEMRA) CD8+ T cells, exhausted CD8+ T cells, and CXCL10+ macrophages—three immune cell types associated with responses to the combination of atezolizumab and bevacizumab, the current standard of care treatment in HCC. In contrast, CD14+ immunosuppressive monocytes are enriched in immune-exhausted tumors, while TREM2+ macrophages are found in both immune-active and immune-exhausted phenotypes. Cancer hallmarks typically associated with each molecular subclass are indicated in the figure. Updated from Llovet et al.^3^

In addition, HCC exhibits additional layers of cellular reprogramming. Mutations in *KEAP1* or *NFE2L2* (Figure 1) rewire HCC metabolism by enhancing antioxidant defenses and supporting anabolic growth, enabling proliferation under oxidative stress and linking metabolic deregulation to the sustained proliferative signaling and evading cell death hallmarks.^6–8^ The convergence of these alterations underpins distinct HCC subclasses^3,41–44^ (Figure 2). These classes show distinct molecular, pathological, and clinical features, and despite not being regularly used in clinical practice, they form the basis for personalized therapies in HCC. About half of HCCs fall into the proliferation class, marked by elevated AFP, poor cell differentiation, *TP53* mutations, 11q13 amplifications, and chromosomal instability.^3^ These tumors have activation of cell proliferation and survival hallmarks, through RAS-MAPK signaling, AKT-mammalian target of rapamycin (mTOR) signaling, and hepatocyte growth factor receptor (MET) signaling^3^—features driving a more aggressive phenotype and poor clinical outcomes. Proliferative tumors include the subtype with non-canonical Wnt/transforming growth factor β (TGF-β) activation, linked to both sustained proliferative signaling and activating invasion and metastasis, as well as the progenitor-like subtype characterized by EPCAM, AFP, and insulin growth factor 2 (IGF2) expression.^13^ Notably, MASH-related HCC shows an increased prevalence of Wnt/TGF-β-driven tumors.^10^ Several genetically driven HCC models have been developed, and they recapitulate the main molecular classes of human HCC. These models capture key molecular and immune features, histopathological appearance, and clinical characteristics including etiology, prognosis, and metastatic potential.^41,45^ Non-proliferation class tumors are described further below.

#### Therapies

Despite the central role of these alterations, direct therapeutic targeting of this hallmark has been challenging. Both *MYC* and *TP53* have been the focus of extensive research yet remain largely undruggable in the clinic. By contrast, the FGF19/FGFR4 axis has emerged as a tractable vulnerability, particularly in tumors with 11q13 amplifications. Several FGFR4 inhibitors, including fisogatinib (BLU-554) and FGF401, have demonstrated activity in early-phase clinical studies, although none has yet advanced to phase 3 trials.^13,46,47^ Finally, CCND1/cyclin-dependent kinase 4 (CDK4) complexes, which are directly inhibited by palbociclib and other CDK4/6 inhibitors, provide another potential therapeutic angle.^48^ Given the frequent co-amplification of CCND1 with FGF19, rational combinations of FGFR4 inhibitors with CDK4/6 blockade warrant consideration as strategies to suppress proliferative signaling in HCC. Finally, several multikinase inhibitors—such as sorafenib, lenvatinib, regorafenib, and cabozantinib—targeting some proliferative pathways (i.e., Ras/MAPK and MET signaling) have been approved for the management of advanced HCC.^3^

### Hallmark: Inducing or accessing vasculature

#### Key molecular alterations

Despite limited progress in targeting the genetic drivers of HCC, progress in understanding the tumor biology has identified features that can be more readily harnessed, with aberrant angiogenesis emerging as one of the best validated. Like many solid tumors, HCC relies on new blood vessel formation to sustain growth and survival, but its pathobiology is distinctive: tumors arise in chronically inflamed and fibrotic livers, environments inherently rich in pro-angiogenic signals.^49^ High-level focal amplifications of 6p21 (locus of *VEGF-A*) are found in 6% of HCC, which correlates with high expression levels. As tumors expand and become hypoxic, hepatocytes and stromal cells upregulate VEGF, which activates phosphatidylinositol 3-kinase (PI3K) and MAPK signaling to promote endothelial proliferation and stromal remodeling. Hypoxia-inducible factor-1 (HIF1) activation and nuclear factor κB (NF-κB)-driven inflammation further amplifies VEGF expression, reinforcing a microenvironment conducive to neovascularization.^50^

#### Therapy

The biological understanding of this hallmark laid the foundation for the development of several targeted therapies in advanced HCC (Figure 1). Sorafenib, originally developed as a Raf kinase inhibitor, also targets VEGFR1/2/3 and other pro-angiogenic kinases, thereby attenuating angiogenesis and improving OS from 7.9 to 10.7 months, compared with best supportive care.^51,52^ Lenvatinib is a dual inhibitor of VEGFR- and pan-FGFRs, which blocks an alternative pro-angiogenic pathway and, as single agent, demonstrated an mOS of 19 months.^13,53^ Regorafenib, a more potent derivative of sorafenib, extends survival post-sorafenib failure and uniquely targets the angiopoietin receptor TIE2, thereby modulating tumor-associated macrophages (TAMs) and broadening anti-angiogenic effects.^54^ Cabozantinib similarly inhibits VEGFR1/2/3 along with MET and TAM family kinases, reducing vascular integrity and permeability.^55^ Rivoceranib (i.e., apatinib) is a highly selective VEGFR2 inhibitor that hinders tumor proliferation and neovascularization, while mitigating TME immunosuppression. In combination with ICIs, OS was significantly improved, compared with sorafenib, for patients with unresectable HCC and has got approval in China.^56^ Beyond tyrosine kinase inhibitors (TKIs), ramucirumab, a monoclonal antibody (mAb) against VEGFR2, provides a benefit for AFP-high patients and is the sole therapy that by blocking angiogenesis selectively achieved survival benefits, highlighting the importance of this hallmark in HCC.^57^

Importantly, angiogenesis blockade reshapes not only tumor vasculature but also immune context. VEGF inhibitors can normalize abnormal vessels, restore micro vessel density, and enhance immune infiltration. They also promote dendritic cell maturation, augment antigen presentation, and activate cytotoxic T cells, while reducing immunosuppressive populations such as TAMs, myeloid-derived suppressor cells (MDSCs), and regulatory T cells (Tregs).^58^ These immunomodulatory properties provide the rationale for combining anti-angiogenic agents with ICIs. Indeed, bevacizumab, an anti-VEGF-A antibody, enhances efficacy when paired with ICIs by simultaneously disrupting tumor vasculature and alleviating immune suppression.^59,60^ The promise of such combinations sets the stage for immunotherapy, now a central pillar of advanced HCC treatment.

### Hallmark: Activating invasion and metastasis

#### Key molecular alterations and subclasses

Metastatic spread is one of the strongest predictors of poor clinical outcomes in HCC. Along with macrovascular invasion, metastatic spread defines the stage C (advanced stage) as per the Barcelona-Clinic Liver Cancer (BCLC) stating system. Prior to the introduction of systemic therapies in 2007, patients in this stage had an mOS lower than 1 year. Among many pathways involved in tumor invasion, aberrant activation of β-catenin has been thoroughly documented in HCC. β-catenin functions both as a transcriptional coactivator in the Wnt/β-catenin signaling pathway and as a structural component for maintaining cell-to-cell adhesion, epithelial integrity, cell polarity, and the overall tissue architecture. In HCC, this structural role is often compromised, as mutations in the *CTNNB1* gene (present 30% of tumors) and inactivating *AXIN1* (~7%) or *APC* (~2%) mutations, which converge to stabilize β-catenin, lead to loss or miss localization of membrane-bound β-catenin.^5,61^ As a result, β-catenin accumulates in the cytoplasm and eventually translocate into the nucleus, where it activates the transcription of genes that promote cell migration, invasion, and epithelial-mesenchymal transition (EMT), contributing to tumor progression and metastasis. Canonical Wnt signaling with *CTNNB1* mutations define a clear molecular subclass within the non-proliferation HCC class (Figure 2).^3,61^ This class is more heterogeneous but generally linked to better prognosis, encompassing well-differentiated tumors enriched for metabolic pathways. These molecular distinctions have direct implications for immunotherapy. For instance, *CTNNB1* mutations that strongly activate the canonical Wnt signaling pathway are typically associated with non-proliferative tumors enriched in the immune-excluded/desert phenotype, characteristically less responsive to immune-based strategies.^62–64^ In contrast, mutations that weakly activate Wnt signaling exhibit greater immune cell infiltration^64^ and are more likely to respond to immunotherapies.

#### Therapy

Despite 30% of HCC having *CTNNB1* mutations, none of the β-catenin inhibitors assessed in early phases of development have advanced to phase 3 investigations. Selective inhibition of the Wnt pathway has shown promising antitumor activity in preclinical models.^65,66^ Early-phase clinical trials (e.g., NCT04008797, NCT06600321, NCT05919264) are now under way to evaluate the therapeutic potential of WNT pathway inhibitors in patients with *CTNNB1*-mutant HCC. Also, recent evidence suggests that β-catenin-mutant HCCs display enhanced dependence on mTOR signaling, identifying a potential metabolic vulnerability. With the advent of dual mTORC1/2 inhibitors, this therapeutic avenue warrants renewed investigation.^45^

### Hallmark: Tumor promoting inflammation

#### Key molecular alterations

HCC emerges not from mutations alone but from their pathological dialogue with the chronically damaged hepatic ecosystem. Chronic liver injury integrates with these molecular features to accelerate malignant transformation. Experimental models show that oncogenes such as *MYC* or β-catenin can induce proliferation but require tissue damage to progress to invasive disease.^67^ When combined with viral proteins, toxins, or dietary stressors, these lesions rapidly advance, underscoring the synergy between intrinsic mutations and extrinsic injury. Cycles of hepatocyte death, regeneration, inflammation, and fibrosis exacerbate genomic instability and provide trophic cues that collaborate with oncogenic pathways.^68^ Collectively, these findings underscore how mutations, gene dosage imbalance, epigenetic deregulation, and tissue damage converge to shape HCC development. Yet most of these alterations do not yield directly druggable targets. While *FGF19-FGFR4* amplification, Wnt deregulation, or *EZH2* overexpression provide therapeutic entry points, the mutational and epigenetic landscape remain challenging to translate into precision oncology.

#### Therapy

Although its role in delaying disease progression remains unclear, there is compelling evidence that reducing liver inflammation significantly lowers the risk of developing HCC. Notable examples include HCC associated with viral hepatitis caused by HCV or HBV. Universal vaccination against HBV in Taiwan resulted in a dramatic reduction in HCC incidence.^3^ Similarly, antiviral therapies targeting HCV and HBV have consistently demonstrated a decrease in HCC risk, although they do not eliminate it.^3^ Regarding the fastest-growing risk factor for HCC-MASH, two FDA-approved medications, resmetirom and semaglutide, have been shown to improve liver fibrosis in patients with stage 2 or 3 fibrosis.^69,70^ While their impact on HCC development remains unknown, their ability to address fibrosis—one of the strongest predictors of HCC—presents a promising opportunity for HCC prevention.

### Hallmark: Avoiding immune detection

#### Key molecular alterations and subclasses

In HCC, immune evasion is particularly pronounced due to the unique immunotolerant environment in which tumors arise. The liver has evolved as a frontline immunological organ that is continuously exposed to gut-derived antigens through the portal circulation, necessitating a high threshold for immune activation.^44^ The mechanisms through which the liver itself exerts a local immunosuppressive program are numerous but may include the secretion of fibrinogen-like protein 1 (FGL1), a liver-secreted protein that is a major functional ligand of the immune checkpoint LAG3.^44^ The HCC TME is typically rich in Tregs, MDSCs, immunosuppressive interferon (IFN)-γ-Tc17 T cells, and M2-polarized TAMs,^44,71^ all of which dampen antitumor immunity. Two dominant immunosuppressive pathways are activated in almost half of the tumors: β-catenin signaling (~30%) and TGF-β signaling (~20%).^3,44^ In addition to the tolerogenic nature of the liver, chronic liver inflammation due to chronic HBV and HCV infection, ALD, and MASH results in sustained antigenic stimulation and promote a state of T cell exhaustion.^41^ Over time, this chronic inflammatory milieu reshapes the immune landscape of the liver in a way that impairs effective tumor surveillance.

While genomic instability in cancer can contribute to immune recognition by generating neoantigens, HCCs are characterized by a relatively low tumor mutational burden (TMB; 2–4 mut/MB), compared with other solid tumors such as melanoma or non-small cell lung cancer.^72^ DNA repair mechanisms in hepatocytes are often relatively intact, further limiting the accumulation of immunogenic mutations. In parallel, because of chromosomal instability and loss of heterozygosity in human leukocyte antigen (HLA) loci, several tumors have the antigen presentation machinery hampered.^13,29,43^ The immunosuppressive nature of HCC results in approximately 30% of tumors being inflamed (“hot”), while around 70% remain non-inflamed (“cold”).^43,44^ Nonetheless, even among inflamed tumors only half of them have been reported to respond to atezolizumab plus bevacizumab, while the other 15% inflamed tumors are less responsive/resistant to these therapies because of the dominance of immunosuppressive cells, such as CD14+ monocytes and TREM2 macrophages, in the TME.^73^

Initial studies using whole-gene expression profiling identified an *immune class* in approximately 25% of HCCs, characterized by high immune infiltration, PD-1/PD-L1 expression, and IFN signaling.^42^ A refined classification using next-generation sequencing defined three major groups: inflamed (37%), intermediate (43%), and excluded (20%, also called immune desert in other solid tumors), with the excluded class enriched in *CTNNB1* mutations and immune evasion features.^43,44,62^ The inflamed class is characterized by high immune infiltration, cytolytic activity, and diverse T cell repertoire. The intermediate class exhibits frequent chromosomal instability with deletions in genes involved in antigen presentation and IFN signaling. This class is significantly enriched in *TP53* mutations. The excluded class presents the lowest immune infiltration of all, with enrichment in *CTNNB1* mutations and PTK2 overexpression due to high level amplifications of 8q24. These immune classes were externally validated using multiplex immunostaining in a cohort of more than 900 samples, which also reported an association between *CTNNB1*-driven molecular subclass and immune exclusion.^43^ Activation of the WNT-β-catenin pathway has gained considerable attention because of its link to an immune-exclusion phenotype in HCC and other tumors.^74^ Tumors with active β-catenin signaling are often described as “immune-excluded,” as several biological mechanisms prevent them from responding effectively to ICIs. However, both inflamed and non-inflamed subtypes of Wnt-β-catenin-mutant HCCs have been identified.^43,74^ Understanding why certain subsets of these tumors may remain susceptible to ICI therapy requires deeper mechanistic studies.

#### Therapy

##### Immune oncology-based treatment combinations. Advanced-stage HCC-approved indications.

**Figure 3.**
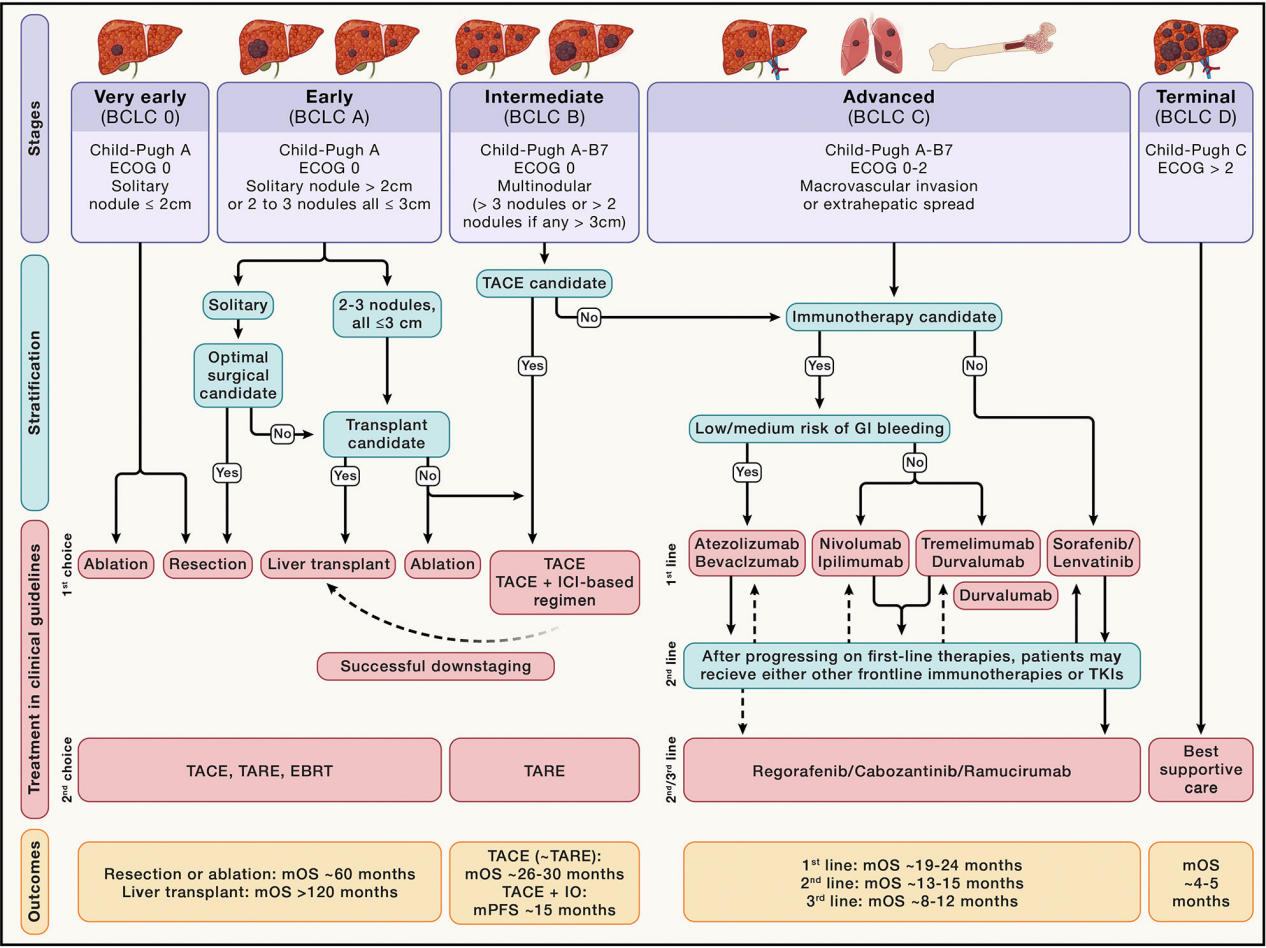
HCC staging system and therapeutic strategy The BCLC system outlines five HCC stages and recommends therapies based on evidence, prioritizing those with proven survival benefit. Resection is advised for patients without clinically significant portal hypertension (CSPH) or with mild CSPH (≤12 mmHg) involving fewer than three segments. If major resection is needed in CSPH, liver transplantation (LT) is preferred. For tumors <3 cm, ablation (radiofrequency or microwave) offers similar efficacy to surgery, although resection allows histological evaluation and early LT planning. Ablation is also suitable for multifocal BCLC-A tumors in non-transplant candidates. Single lesions <8 cm may qualify for curative treatment. First-line treatments for patients at advanced stages include combinations like atezolizumab-bevacizumab, durvalumab-tremelimumab, and nivolumab-ipilimumab; these may enable curative reassessment in complete responders. Solid arrows in the figure indicate treatments with strong evidence; dotted arrows denote investigational second-/third-line options. OS benefit post-sorafenib has been shown in phase 3 trials. Abbreviations: BCLC, Barcelona Clinic Liver Cancer; ECOG, Eastern Cooperative Oncology Group; TACE, transarterial chemoembolization; TARE, transarterial radioembolization; EBRT, external beam radiation therapy; LT, liver transplantation; IO, immuno-oncology; OS, overall survival; CR, complete response; mPFS, median progression-free survival; mTTP, median time to progression. Adopted from Mauro et al.^12^

The development of ICIs has reshaped the management of HCC, and immune-based combinations are currently the backbone for frontline systemic therapies in HCC (Figure 3). One breakthrough involved the phase 3 trial IMbrave150 that combined atezolizumab, a PD-L1 immune checkpoint inhibitor, with bevacizumab, a drug that blocks blood vessel growth. This combo significantly improved patient survival and response rates, establishing a new standard for first-line treatment in advanced HCC. VEGF, a key factor in blood vessel formation, also suppresses immune responses by impairing immune cell function within tumors. Blocking VEGF can normalize tumor blood vessels, improve drug delivery, and enhance immune cell infiltration, making immunotherapies more effective. Another promising approach targets multiple immune checkpoints simultaneously, such as combined PD-1/PD-L1 and CTLA-4 checkpoint blockade, aiming to enhance T cell priming and expand the breadth of the antitumor immune response. This was evaluated in the HIMALAYA trial and demonstrated improved survival without a significant increase in toxicity.^75^ Similarly, the CheckMate 9DW trial evaluating nivolumab (a PD-1 inhibitor) plus ipilimumab (a CTLA-4 inhibitor) showed improved survival over sorafenib, with the highest response rate ever reported in advanced HCC (36%).^76^

There are notable differences between these two approaches. They have distinct priming strategies: the HIMALAYA trial used a single dose of CTLA-4 inhibitor, whereas CheckMate 9DW used four doses. They also differ in their antibody type (immunoglobulin G [IgG] subclass) and how strongly they bind Fc gamma receptor (FcγR).^77^ In conclusion, the evolution of immunotherapy in HCC reflects a strategic targeting of multiple cancer hallmarks,^6–8^ including immune evasion, angiogenesis, and sustained proliferative signaling. Together, these strategies represent a mechanistically grounded effort to expand the therapeutic arsenal in advanced HCC.

##### Early stages.

The immune evasion and angiogenesis hallmarks have been concurrently targeted in this setting to prevent recurrence following curative treatments such as surgical resection or ablation (Figure 3). The phase 3 IMbrave050 trial^78^ tested atezolizumab combined with bevacizumab in HCC patients at high risk of recurrence following surgery or ablation. Despite an early signal of efficacy, mature data did not demonstrate improvement in recurrence-free survival. Similarly, the KEYNOTE-937 trial comparing pembrolizumab with placebo after resection also failed to improve patient’s recurrent-free survival. A proposed promising approach is to use both neoadjuvant therapy (prior surgery) along with after surgery (adjuvant therapy).^79^ Mechanistically, in the neoadjuvant setting, immunotherapy uses the presence of the full, intact tumor as a rich source of antigens, which helps to prime and expand preexisting tumor-reactive T cells. Early proof-of-concept phase 1b-2 trials testing ICI alone or in combination with TKI in this setting showed promising results.^80–82^ For example, in patients treated with the PD-1 inhibitor cemiplimab before surgery, a significant tumor necrosis (defined as >70%) was reported in up to 35% of patients.^81^ More recently, the CARES 009 phase 3 trial^83^ has provided additional support for the idea that perioperative immunotherapy can improve outcomes in these patients. However, these findings still need to be confirmed in other populations and with a longer follow-up.

##### Intermediate stages (BCLC-B).

Locoregional therapies such as transarterial chemoembolization (TACE) work both by blocking the blood supply and delivering a high dose of chemotherapy directly to the tumor (Figure 3). Recent phase 3 trials, LEAP-012^84^ (TACE was combined with lenvatinib and pembrolizumab) and EMERALD-1^85^ (durvalumab and bevacizumab in combination with TACE), have shown that adding systemic therapies to TACE significantly delayed progression-free survival, compared with TACE alone. These data did not translate into significant benefits in OS, but they reinforce the concept that combining systemic and locoregional therapies delays tumor progression and plays a role in the management of these patients (Figure 3).

##### Emerging targeted immunotherapeutic strategies.

ICI have markedly advanced the treatment of HCC with objective responses to current doublet regimens of 20%–36% and durable remissions or cures in the setting of advanced disease with actuarial 4–5-year survival rates of ~20%–25%.^86^ However, immune-related adverse events (irAEs) continue to pose a substantial clinical challenge. These toxicities result from a loss of peripheral tolerance and immune activation against healthy tissues and require high-dose corticosteroid intervention in approximately 10% of patients receiving PD-1/PD-L1 monotherapy and 20%–30% of those treated with dual checkpoint blockade.^76^ To further enhance immune-mediated antitumor activity in HCC and counteract immune destruction evasion, several key strategies are being developed/tested: (1) integrating immune-based therapies at earlier stages of the disease, including in the neoadjuvant setting; (2) incorporating additional compounds targeting novel immune checkpoints; (3) utilizing cancer vaccines based on personalized neoantigen burden; (4) utilizing cell therapies, such as chimeric antigen receptor (CAR) T cell therapies, to target HCC antigens or T cell receptor (TCR)-engineered T cells; (5) utilizing new therapeutic approaches, such as bispecific antibodies, antibody drug conjugates (ADCs) or radiopharmaceutical therapies; (6) targeting other modulators of the TME like cancer-associated fibroblasts (CAFs) through, for example, TGF-β inhibition; and (7) employing biomarker-driven treatment allocation (Figure 1).

Attempts to intensify immunotherapy by adding a third ICI to existing regimens have thus far failed to improve efficacy.^87^ Despite encouraging results in early trials (Morpheus program), the addition of new checkpoint inhibitors (e.g., the anti-TIGIT antibody tiragolumab) to atezolizumab and bevacizumab did not add significant clinical benefit in large confirmatory trials.^87,88^ Novel combinations testing PD-1 checkpoint inhibitors and TGF-β inhibitors are ongoing.^89^ Due to the prevalence of immune-related treatment adverse events, the concept of “targeted immunotherapy” has emerged. This approach aims to selectively activate antitumor immunity while minimizing systemic immune toxicity.

Personalized tumor-derived neoantigen vaccines (PTCVs) prime T cells against tumor-specific mutations not subject to central tolerance. Neoantigen-specific immunity is thought to drive much of the clinical activity of checkpoint blockade, and vaccination aims to enhance these responses and further combat immune destruction evasion. Since neoantigens differ across patients, most PTCVs are individualized, requiring tumor sequencing, neoantigen prediction, and custom vaccine production—historically challenging because of costs and logistics. However, falling sequencing costs and flexible vaccine platforms have made this more feasible. A first-in-human study in HCC evaluated a personalized DNA plasmid vaccine (GNOS-PV02) encoding up to 40 neoantigens, co-delivered with plasmid interleukin (IL)-12 and pembrolizumab in TKI-treated patients. This phase 1/2 trial reported higher response rates (30%) than expected with anti-PD-1 monotherapy. These findings demonstrate proof of concept for personalized vaccination in HCC and suggest vaccines may boost immune responses in less immunogenic tumors.^90–92^

Beyond vaccines, multiple other targeted immunotherapies are under active investigation in HCC, including CAR T cells, bispecific antibodies, and TCR-engineered T cells. The major barrier that these strategies have encountered has been the lack of a uniformly expressed, truly tumor-specific surface antigen. AFP, presented in the context of HLA-A*02:01, has been targeted by TCR-based therapies but with limited clinical efficacy.^93^ Glypican-3 (GPC3), a membrane-bound heparan sulfate proteoglycan overexpressed in many HCCs, has emerged as a leading target for CAR T cell development. Early-phase trials of GPC3-directed CAR T cells have shown initial signs of safety and activity,^94^ and larger studies are ongoing to evaluate clinical benefit. Finally, oncolytic viruses represent another modality with unique relevance to HCC.^95^ These agents selectively replicate within tumor cells, leading to direct lysis and release of tumor antigens, while also triggering innate and adaptive immune responses. Some oncolytic viruses additionally encode immune stimulatory transgenes (e.g., granulocyte-macrophage colony-stimulating factor [GM-CSF] or IL-12) to further enhance immune priming. HCC may be particularly amenable to intratumoral viral therapy given the extensive clinical experience with liver-directed interventions (e.g., TACE, ablation), allowing for reliable intralesional delivery. So far, early-phase trials of oncolytic viruses in HCC suggest limited immunologic activity with manageable safety profiles.^96^ These and other approaches continue to mature with the goal of more precisely activating the immune system against liver cancer, while minimizing systemic toxicity.

### Biomarkers of treatment response

#### Landscape and challenges

##### Landscape.

Accurate treatment allocation is essential to avoid unnecessary toxicity in non-responders and enables curative strategies for likely responders. In HCC, only one biomarker is currently recommended by clinical guidelines; ramucirumab^97^ is recommended in the third-line setting for patients with AFP levels exceeding 400 ng/mL. Other biomarkers proven to be predictive in other cancers, such as TMB or PD-L1 expression, are not significantly associated with responses in HCC. Although HCC lacks a structured framework for selecting patients, some associations with treatment response have been described. For TKIs, sorafenib appears more effective in patients with HCV and without metastases.^98^ Specific plasma markers (including ANG-1, cystatin B, LAP, TGF-β1, OLR1, and CCL3) were linked to longer survival after regorafenib treatment, suggesting their potential as predictive biomarkers.^99^ For single-agent ICIs, transcriptomic signatures identifying inflamed HCC tumors have been linked to better responses to anti-PD-1 monotherapy,^42,43,100^ including an 11-gene signature related to IFN-γ signaling and antigen presentation.^100,101^ For ICI-based combinations, preexisting immunity (high PD-L1, effector T cell signatures, and CD8+ T cell infiltration) correlated with better outcomes in patients receiving atezolizumab plus bevacizumab.^60^ Single-cell analysis suggest distinct response patterns, immune-driven and angiogenesis-driven, and implicate immunosuppressive myeloid cells and Notch/TGF-β signaling in resistance to atezolizumab plus bevacizumab.^73^ These findings warrant validation for clinical implementation.

##### Challenges.

Several factors contribute to the absence of predictive biomarkers in HCC: (1) lack of prevalent *bona fide* drug-gable mutations. (2) Challenges in tissue acquisition, as non-invasive imaging-based HCC diagnosis is achieved in over 80% of cases who meet appropriate criteria.^97,102^ (3) Marked tumor heterogeneity. While truncal mutations are shared across distinct tumor sites or metastases, HCC exhibits significant intertumoral and intratumoral variability, compounded by a complex etiological background including cirrhosis due to viral hepatitis, metabolic syndrome, and ALD. Efforts are shifting toward single-cell transcriptomics, proteomics, and epigenetic profiling to deepen our understanding of cellular signals that might enable the discovery of biomarkers. Also, non-invasive biomarkers (e.g., circulating tumor DNA,^103^ deep learning-based image-based predictors^104^) may help overcome the limitations of accessing HCC tissue samples for molecular profiling include.

#### Biomarker-based clinical trials

The development of ICI has revolutionized oncology and reshaped the design of clinical trials. Unlike traditional therapies, ICI often elicit delayed but durable responses in a subset of patients, necessitating the adoption of alternative measures to assess clinical benefit.^105,106^ Precision oncology has been implemented in early-phase trials, selecting patients based on oncogene addition FGF19 or *CTNNB1*, with suboptimal signals to move forward with phase 3 pivotal studies.^107^ Nowadays, oncology trial designs have become more adaptive and biomarker-driven, incorporating enrichment strategies, basket trials, and platform trials that enable real-time learning and cohort expansion based on biomarker-defined subgroups. The Morpheus trial program (NCT032137119), GEMENI, and the NCI-MATCH^108^ initiative exemplify this approach.

### Insights into the therapeutic implications of cancer hallmarks in HCC

Clinical practice guidelines (CPG) recommend the BCLC staging and treatment algorithm as the standard framework for the management of HCC^97^ (Figure 3). This system emphasizes a multi-disciplinary approach and integrates data from tumor burden, liver function, and performance status to classify patients into five different stages. Potential curative options for patients at very early (BCLC-0; single tumor less than 2 cm in diameter) or early (single tumor or up to three nodules, none larger than 3 cm) stages include resection, ablation, and liver transplantation. Patients at this stage have mOS beyond 60 months. Intermediate-stage patients include patients with multiple liver nodules but without macrovascular invasion or extrahepatic spread. They are typically managed with transarterial therapies, including chemoembolization or yttrium-90-based radioembolization, which yields mOS of 26–30 months. Downstaging, defined as the intentional reduction of tumor burden through locoregional therapies, with the goal of bringing the disease within acceptable criteria for transplantation is widely accepted. In some patients where tumor burden is extensive or those with infiltrative tumors, systemic therapies can be considered. Patients at advanced stages, defined by the presence of macrovascular invasion or extrahepatic metastasis (generally to the lung, bone, or lymph nodes), are candidates for systemic therapies. Immune-based combination therapies are the backbone frontline systemic therapy in HCC, as previously described. TKI is reserved for patients who have contraindications (e.g., autoimmune disease), are intolerant, or progress to immune-based therapies. This includes sorafenib and lenvatinib, while cabozantinib or regorafenib are generally recommended upon progression of sorafenib or lenvatinib.^12^ Ramucirumab, a mAb targeting VEGFR2, increases OS in patients with AFP ≥400 ng/mL in the third-line setting after a biomarker-design clinical trial.^57^

Terminal-stage patients (BCLC-D) mainly because of poor performance status or severe liver dysfunction should receive the best supportive care.

Understanding the Hallmarks of Cancer^6–8^ has greatly influenced HCC therapies (Figure 1), which traditionally responded poorly to conventional chemotherapy. Targeting the hallmarks “sustained proliferative signaling,” “inducing or accessing vasculature,” and “avoiding immune detection” with TKIs, mAbs like bevacizumab, alongside ICIs, have significantly improved OS in HCC. These advances have almost tripled the lifespan of patients with HCC in the past two decades, from a median of ~8 months (prior to the seminal SHARP trial^51^) to the current 20–24 months with immune-based combination therapies.^59^ Expanded hallmarks, like altered metabolism and inflammation, are creating new therapeutic opportunities, including targeting the TME. Progress in early or intermediate stages has been limited, with treatment still primarily relying on surgical resection, transplantation, or transarterial therapies. Early data from phase 3 trials suggest that systemic therapies may also be effective in combination with these therapies.^109^ Integrating emerging biomarkers like gene expression, immune signatures, and circulating tumor DNA will enhance patient stratification and therapeutic response monitoring, supporting adaptive clinical trials that improve efficiency and outcomes.

### iCCA

iCCA represents the second most common primary liver cancer after HCC, accounting for ~10%–15% of liver cancer cases. iCCA typically arises from the small bile ducts within the liver, and despite being traditionally considered a cholangiocyte neoplasm, emerging evidence shows that it may also originate from the hepatocyte through trans-differentiation. Aside of iCCA, there is evidence that hepatic progenitor cells, their intermediate states, or de-differentiated hepatocytes can form liver tumors with progenitor-like features, including mixed HCC-CCA and cholangiolocellular carcinoma.^16,110–112^ Well-established risk factors for iCCA include chronic liver injury and inflammation, such as primary sclerosing cholangitis, biliary cysts, and fluke infections.^113^ Factors linked to HCC (i.e., chronic HBV and HCV infections, cirrhosis, etc.) also increase iCCA risk, yet most cases (60%–70%) remain idiopathic.

iCCA has an age-standardized incidence rate of 1.4 per 100,000 person-years,^113,114^ representing 3% of all gastrointestinal malignancies. Despite its low prevalence, incidence has steadily risen, with a 128% increase in the US between 1973 and 2012, and continues to rise.^115^ This rise, combined with the high mortality,^116^ underscores the ongoing challenge of optimal management. Albeit rare in the Western world, iCCA represents the most common liver cancer in Thailand, where endemic liver fluke infections drive incidence rates up to 80 per 100,000 people annually.^113,117^

**Figure 4.**
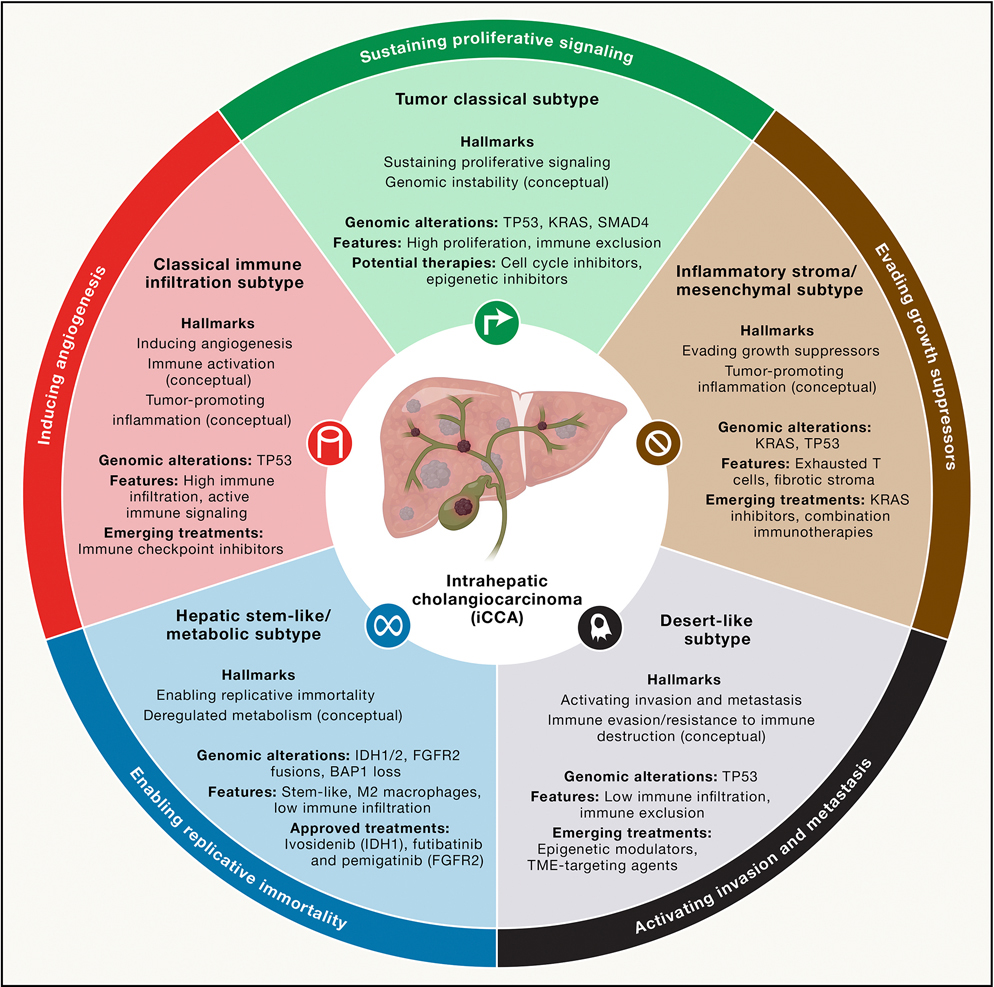
Cancer hallmarks, molecular classes of iCCA, and therapies Major hallmarks of cancer as they relate to iCCA, highlighting the key molecular subclasses of the disease and associated therapeutic strategies. Molecular alterations commonly observed in iCCA—such as FGFR2 fusions, IDH1/2 mutations, BAP1 loss, and *KRAS*/*TP53* mutations—are mapped to their corresponding hallmarks, including inducing angiogenesis, evasion of growth suppressors, enabling replicative immortality, and activating invasion and metastasis. The figure further categorizes current and emerging targeted therapies, immunotherapies, and conventional treatments according to the molecular class they are designed to target, underscoring the precision medicine approach in iCCA management.

Early diagnosis of iCCA is challenging since patients generally present with mild, non-specific symptoms, with most cases diagnosed at advanced stages when potentially curative options are limited. Therefore, prognosis is poor, with mortality rates paralleling incidence rates (1–2/1,000,000 people)^116^ and 95% of patients dying within 5 years.^118^ For advanced disease, the first-line therapy is gemcitabine and cisplatin plus durvalumab or pembrolizumab, achieving an mOS of approximately 13 months.^119,120^ Over the past decade, massive parallel sequencing has revolutionized iCCA management in patients, with the discovery of actionable alterations, belonging to different hallmarks in ~45% of patients (Figure 4; Table 2), paving the way for precision oncology.

### Hallmark: Sustaining proliferative signaling

#### Key molecular alterations

iCCA exhibits a highly heterogeneous mutational landscape. The most frequent mutations found in iCCA occur in genes able to maintain self-sufficient growth such as *KRAS* (~22%), *FGFR2* fusions (~10%–15%), *PIK3CA* (~8%), *BRAF* (~5%), and ERBB2 (1%–2%).^121–123^ Activation of the fibroblast growth factor pathway is common in cancer. The pathway consists of 4 receptors (FGFR1–4) and 18 ligands.^124^ Research-based studies have reported that ~25%–30% of iCCAs present FGFR2 activation mostly via gene fusions,^122,125–129^ but it can also be affected through rearrangements, point mutations, or in-frame deletions.^121^ Notably, a lower percentage (10%–15%) has been subsequently reported in clinical studies, probably because of the suboptimal sequencing technologies employed in the clinical setting to identify rearrangements. *FGFR2* fusions promote ligand-independent FGFR dimerization and activation, driving cellular transformation, likely through loss of an inhibitory domain in the final exon of *FGFR2*.^130^ Besides *FGFR2*, another signaling pathway that plays a key role in sustaining proliferative signaling in iCCA is ERBB2. While *ERBB2* mutation rate is low (1%–2%), amplification/overexpression has been reported in about 5% of iCCA.^131^

Overexpression and activation of CDKs, particularly CDK1, CDK2, and CDK4/6, are common in this tumor.^132^ *PI3K/AKT/mTOR* pathways, which intersect with cell-cycle control, are also upregulated in iCCA and represent additional avenues for targeted therapy, with preclinical data supporting their potential utility.^133^ Multi-CDK inhibitors like roscovitine induce cell-cycle arrest, inhibit proliferation, and promote apoptosis in CCA models, with *in vivo* efficacy demonstrated in xenograft systems.^132^ However, CDK4/6 inhibitors (i.e., palbociclib) as a single therapy showed lack of efficacy in early phase 2 clinical trials in iCCA, and no CDK inhibitors are currently approved for iCCA outside of clinical trials.

Downstream of receptor tyrosine kinase growth factor activation, *Ras/MAPK* pathway signaling is also an established target across multiple tumor types. Activating mutations in the *KRAS* gene is among the most frequent genomic alterations in iCCA (~22%, Table 2), with the hotspot^*G12D*^ being the most frequent (~50% of all *KRAS* mutations) followed by other changes in codon 12 (^G12V^ and ^*G12C*^, 31%), whereas up to 5% of iCCAs harbor *BRAF*^*V600E*^ mutations.^134^ Notably, KRAS is a potent oncogenic driver whose effects extend beyond the sustained proliferation property conferred to tumoral cells and vary from modulation of CAFs to immunosuppression of the antitumor immune response. CAFs are the most abundant cell type in the iCCA stroma and the main secretors of the extracellular matrix (ECM). The extent of desmoplasia^135^ and the abundance of activated CAFs (measured as expression of αSMA)^136^ as well as other ECM-related factors (i.e., LOXL2)^137^ have been closely associated with worse outcomes in iCCA. The application of single-cell technologies in human iCCA specimens has revealed at least four transcriptomic distinct subtypes: ECM-related myofibroblastic CAFs (myCAFs), inflammatory CAFs (iCAFs) enriched in cytokines and growth factors, antigen-presenting CAFs (apCAFs), and vascular CAFs (vCAFs).^138,139^ In other cancer types, CAFs play dual roles that can either promote or delay tumor growth, but in iCCA, evidence mainly point toward a sustained “tumor-promoting role.”^138,139^

#### Therapy

Most FDA-approved treatment strategies for iCCA patients target this critical hallmark. The FGFR1–3 inhibitors, pemigatinib and infigratinib, were the first to receive FDA and EMA approval for iCCA patients with *FGFR2* fusions, based on positive high-response rates in early-phase clinical trials.^126,140^ However, infigratinib confirmatory trials were terminated early because of feasibility challenges, leading the sponsor to voluntarily withdraw the agent. Acquired resistance to these therapies, due to mutations in the *FGFR2* kinase domain, has spurred the development of irreversible kinase domain inhibitors.^141^ The irreversible kinase domain inhibitor, futibatinib, also obtained accelerated FDA and EMA approval.^142^ Next-generation irreversible FGFR2 fusion inhibitors like lirafugratinib are currently being tested in clinical trials. Additional efforts focus on combination therapies targeting^123,126^ non-mutational adaptive pathways (e.g., EGFR signaling), antagonistic bi-paratropic antibodies (recognizing two extracellular FGFR2 epitopes), immunotherapy,^143^ and next-generation kinase inhibitors active against common resistance mutations. Besides FGFR2, another clinically relevant alteration in iCCA is ERBB2 overexpression. The bispecific anti-body, zanidatamab, achieved objective responses in 52% of biliary cancer patients with ERBB2 overexpression (174).^144^ The antibody-drug conjugate trastuzumab deruxtecan showed similar objective response rates and received tumor-agnostic accelerated approval. There is also response rates of 42% in biliary cancers with KRAS^G12C^ mutations treated with selective inhibitors such as adagrasib.^134,145,146^

### Hallmark: Deregulating cellular metabolism

#### Key molecular alterations

Metabolic enzymes such as IDH1–3 catalyze the oxidative decarboxylation of isocitrate to α-ketoglutarate (αKG). IDH1 and IDH2 can be mutated as gain-of-function variants that increase production of R-2-hydroxyglutarate (2HG). This aberrant metabolite accumulates in the cell and inhibits αKG-dependent enzymes interfering with cell metabolism and epigenetic gene regulation.^147^ *IDH1* and *IDH2* mutations are observed in approximately 20% and 5% of iCCAs,^121^ respectively, as well as in glioma, acute myeloid leukemia, and chondrosarcoma. The most common *IDH1* mutant allele is R132H and for *IDH2* is R140Q.

#### Therapy

Inhibitors have been developed for each enzyme, namely, ivosidenib for IDH1 and enasidenib for IDH2. Only the IDH1 inhibitor ivosidenib secured FDA and EMA approval for treatment of iCCA, as it significantly improved progression-free survival in the phase 3 clinical trial.^148^ The activity of ivosidenib is primarily cytostatic, and duration of response remains a critical challenge. Primary and secondary resistance mechanisms are incompletely understood and remain a focus of active investigation.

### Hallmark: Genome instability and mutation

#### Key molecular alterations

Genome instability generally results from mutations (either germline or somatic) in DNA repair genes. This impairs the ability of the cell to fix DNA damage, leading to the accumulation of mutations or other genomic changes, including microsatellite instability (MSI). A high number of somatic mutations (measured as TMB) in MSI-high or deficient mismatch repair (dMMR) tumors, as assessed by either polymerase chain reaction or immunohistochemistry, lead to the production of high-quality neoantigens, increasing T cell reactivity and improving response to ICI.^149^ MSI rarely occurs in iCCA, with a reported prevalence of 0.5%–6%.^122,150–153^ Similarly, TMB levels are modest in iCCA with a median of 2 mut/MB^154^ and only a small fraction of patients identified as harboring a TMB greater than 10 mut/MB.^121,155^ *BRAC1/2* genes are also involved in DNA repair, but mutations are rare in iCCA (1%–3%). Aside from the mutations in genes linked to the hallmark “sustained proliferation,” others frequently found in iCCA include *TP53* (~27%), *CDKN2A/B* (~27%), *ARID1A* (~18%), *BAP1* (~10%–15%), and *SMAD4* (~10%).^121,122^ This mutational diversity reflects both clonal and sub-clonal evolution, contributing to intratumoral heterogeneity, variable response to treatment, and resistance to standard therapies.^117^ While TMB is mostly unremarkable, several broad DNA copy-number gains in iCCA have been described, involving chromosomes 1q and 7p (~25%), as well as several broad losses. In addition, high-level focal chromosomal amplifications have been reported, for instance, at 11q13.2 (involving CCND1, FGF family members, and ORAOV1) and 1p13.1 (spanning several transcriptional repressors, e.g., TRIM45, TTF2, and VTCN1; Table 2).^4^

#### Therapy

The presence of dMMR and/or TMB (= or >10 mut/Mb) is clinically significant, as the FDA has granted tumor agnostic approval of pembrolizumab for patients with metastatic MSI-high or dMMR solid tumors.^156^ On the other hand, *BRAC1/2* mutations confer sensitivity to targeted therapies such as PARP inhibitors in several solid tumors, including breast, ovarian, and pancreatic cancer,^157^ suggesting a potential role for future studies of PARP inhibitors in iCCA patients with *BRAC1/*2 mutations or other alterations associated with homologous recombination repair deficiencies.^158^ Other mutations linked to this hallmark remain currently undruggable.

### Hallmark: Avoiding immune detection

#### Key molecular alterations

Approximately half of the iCCAs exhibit low lymphocytic infiltrates and a high prevalence of immunosuppressive signals, facilitating immune evasion and resistance to immune-mediated destruction.^159–161^ A well-known mechanism to evade immune surveillance involves the expression of immune checkpoint molecules. While PD-L1 expression may identify patients likely to benefit from anti-PD-1/PD-L1 blockade, it has become obvious that its quantification alone is inadequate to predict tumor response in most malignancies,^162^ including iCCA.^119,163,164^ Overall, PD-L1 protein expression (>1% of cells) in iCCA remains low (2%–4%) and is mostly restricted to TAMs rather than cancer cells,^165^ which is consistent with the relatively low clinical efficacy of anti-PD-1 therapies as single agents in iCCA.^163,166,167^ Other immune checkpoint molecules, such as PD-1, CTLA-4, and LAG3, exhibit heterogenous expression within CD4+ and CD8+ T cells^168,169^ and across tumor location (center vs. periphery).^161^ Notably, high PD-1 or LAG3 expression and low CD3/CD4/ICOS at the center of the tumor appear to significantly correlate with low OS in iCCA.^161^ The therapeutic implications of these findings remain unclear. Newly recognized immune checkpoint molecules, such as B7-H3 and B7-H4, have been detected at the protein level in a substantial proportion of iCCA patients,^161,170–172^ particularly those harboring *BAP1* mutations.

#### Therapy

A combination of anti-PD-1/PD-L1 with chemotherapy has recently become the standard of care for advanced iCCA.^119,120^ Only one-third of patients respond, regardless of their tumor PL-D1 status.^119,120^ PD-L1 intratumoral heterogeneous expression could explain its inadequacy as a biomarker. In this regard, a model of spatial hallmark patterns and their utility to predict therapeutic responses has been recently proposed based on data from 10 tumor types,^173^ including HCC and iCCA. Several clinical trials are testing therapeutics aimed at targeting B7-H4 including mAbs, CART T cells, and antibody-drug conjugates. So far, trials investigating inhibitory anti-B7-H4 mAbs failed to show any significant clinical benefit. The limited activity of mAbs is not surprising given emerging evidence pointing to a T cell-independent role of B7-H4 in iCCA.^171^ In summary, iCCA commonly evades antitumor immune responses, making ICIs less effective. However, when combined with chemotherapy, ICIs can enhance survival, highlighting the need for a deeper understanding of the interplay between cancer cells and the TME.

### Integrating key iCCA hallmarks into a new molecular classification system

**Figure 5.**
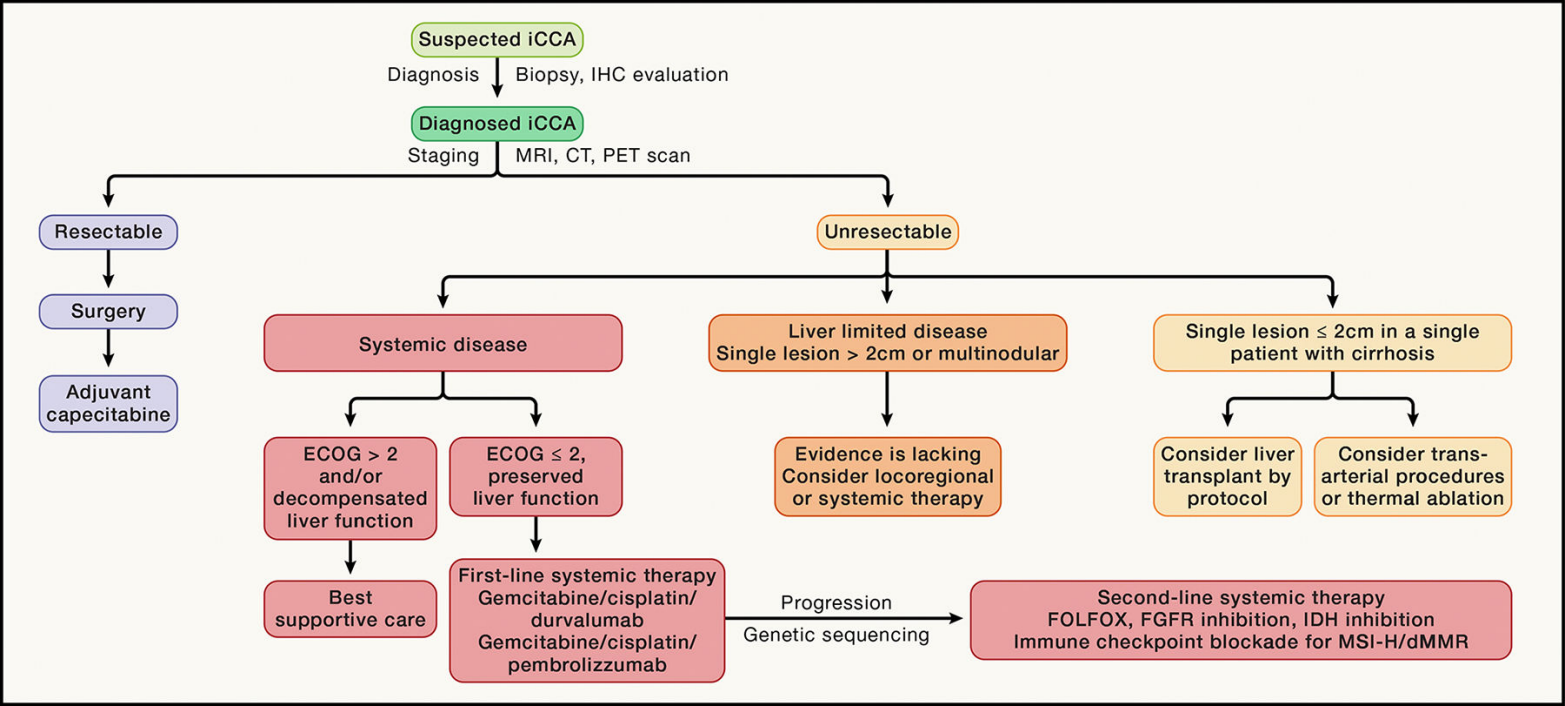
Diagnosis and management of iCCA ^†^Biopsy could be avoided in resectable suspected iCCA since definitive histopathological confirmation can be obtained in the surgical specimens. *For patients harboring these targetable mutations. Abbreviations: FGFR, fibroblast growth factor receptor; FOLFOX, oxaliplatin/fluorouracil; iCCA, intrahepatic cholangiocarcinoma; IDH, isocitrate dehydrogenase; PET, positron emission tomography. Source: EASL-ILCA CPG on the management of intrahepatic cholangiocarcinoma.^113^

To enhance patient and therapeutic stratification in iCCA, various molecular classification systems have been proposed over the past two decades. While initial classifications focused on intrinsic characteristics of cancer cells^117,174^ or histological/anatomical features (small-duct and large-duct iCCA),^175^ contemporary ones integrate mutational profiles and the TME, offering a comprehensive view of tumor biology and therapeutic opportunities.^159,160,176–181^ Broadly, iCCA can be classified into classical immune infiltration, inflammatory stroma or mesenchymal, desert-like, hepatic stem-like or metabolic, and tumor classical (Figure 5). The classical immune infiltration subtype is characterized by high immune infiltration and active immune signaling, aligning with the hallmarks of immune activation and tumor-promoting inflammation. These tumors show frequent *TP53* mutations and are predicted to respond well to ICIs, making them prime candidates for immunotherapy.^126,160,161,177,181^ In contrast, the inflammatory stroma or mesenchymal subtype exhibits infiltration with exhausted T cells and abundant fibrotic stroma. Frequently associated with *KRAS* and *TP53* mutations, this subtype reflects hallmarks such as immune evasion and tumor-promoting inflammation. Therapeutic strategies may include *KRAS* inhibitors and combination treatments targeting both immune exhaustion and the TME.^159,160,177^ The desert-like subtype is enriched in *TP53* and *KRAS* mutations and defined by immune exclusion. These tumors are unlikely to benefit from immunotherapy alone and may require epigenetic modulators or TME targeting.^159,160,177^ The hepatic stem-like or metabolic subtype features a stem-like phenotype and M2 macrophage enrichment. It exhibits *IDH1/2* mutations, *FGFR2* fusions, and *BAP1* loss and is characterized by low immune infiltration, aligning with cancer hallmarks^6–8^ such as epigenetic reprogramming and deregulated metabolism.^160,176,181^ This group is responsive to targeted therapies, including IDH1 (ivosidenib) and FGFR inhibitors (pemigatinib, futibatinib). Notably, emerging evidence shows that the presence of FGFR2 fusion correlates with accumulation of granulocytes,^182^ although underlying mechanisms remain poorly understood. Whereas preclinical evidence in animal models suggests that mutant IDH inhibits TET2-mediated DNA demethylation and thus suppresses IFN-γ signaling, leading to immune evasion.^183^ These pieces of evidence suggest that immunotherapeutic combinations targeting these specific immune vulnerabilities could represent more appealing strategies. Finally, the tumor classical subtype is enriched in *TP53*, *KRAS*, and *SMAD4* mutations and exhibits high proliferative activity, genomic instability, and immune exclusion. It reflects hallmarks of sustained proliferative signaling and genomic instability, and these tumors may benefit from chemotherapy, cell-cycle inhibitors, or epigenetic modulators. Although this classification is not routinely applied in clinical practice, it illustrates the variability in the dominance of cancer hallmarks in iCCA and underscores the importance of understanding their relative contributions within individual tumors to guide optimal treatment allocation strategies.

### Insights into the therapeutic implications of cancer hallmarks in iCCA

The diverse array of candidate molecular targets (Figures 4 and 5; Table 2) in both the tumor and microenvironment of iCCA has ushered in a new era of molecularly targeted therapy. In the first-line setting for advanced stages of disease, targeting the hallmarks of sustained proliferative signaling, “genome instability and mutation,” and avoiding immune detection with combined chemotherapy and ICI have become standard treatment based on successful clinical trials.^119,120^ The phase 3 TOPAZ-1 and KEYNOTE-966 trials showed that adding durvalumab or pembrolizumab to the previous standard of care of gemcitabine-cisplatin chemotherapy improved OS, compared with chemotherapy alone. Although improvements were modest overall, approximately 25% of patients achieved survival beyond 24 months in each trial, substantially exceeding the historical 10% benchmark.

These improved outcomes may be attributed to several potential mechanisms, including enhanced antigen presentation and major histocompatibility complex (MHC) class 1 expression with gemcitabine exposure, chemotherapy-mediated inhibition of immune-suppressive components of the TME, or an inflammatory immune infiltration induced by platinum-mediated ferroptosis. After progression on first-line therapy, standard chemotherapy regimens confer limited benefit to most patients. In this context of significant unmet need, a host of molecularly targeted agents have demonstrated promising activity leading to clinical uptake in subsets of iCCA after progression on frontline therapy (Table 2). The most successful examples of second-line targeted therapies include the previously described *FGFR2* and *IDH* inhibitors and the mAbs against ERBB2, which target the hallmarks “sustained proliferative signaling” and “deregulating cellular metabolism.”^6–8^ Reinforcing the relevance of ERBB2, cholangiocarcinoma patients harboring *NRG1* fusions—a ligand for the HER family—demonstrated responses to the ERBB2/HER3 bispecific antibody, zenocutuzumab, in a phase 2 basket trial that led to tumor-agnostic regulatory approval in the US.^184^

Promising clinical activity in advanced iCCA, along with relatively high rates of recurrence after resection, supports investigation of new molecularly targeted agents in earlier stages of disease, and several clinical trials are ongoing in the perioperative and adjuvant settings for a variety of molecular subsets (Figures 4 and 5; Table 2). Enrollment to these trials has been hampered, however, by the extreme rarity of individual molecular aberrations, the infrequent candidacy of newly diagnosed iCCA patients for surgery or locoregional therapy, scarce tumor tissue for profiling, and the urgency of a grim prognosis that often prompts treatment initiation before molecular profiling results are available. At present, there is no established role for molecularly targeted agents in the perioperative or adjuvant settings.

Given the emerging “sustaining proliferative signaling” role of CAFs, several strategies have tried to target this population in iCCA, but none is currently FDA-approved. Broad CAF elimination with navitoclax^185^ or those aimed at suppressing CAF activation with TGF-β inhibitors have been tested preclinically and clinically with limited success.^186^ More promising strategies focus on targeting specific CAF markers, particularly tumor-promoting receptor-ligand interactions between CAFs and cancer cells, such as hepatocyte growth factor (HGF)-MET signaling (iCAF-mediated), hyaluronan (HA)-HA synthase (myCAF-mediated), and IL-6/IL-6 receptor (vCAF-mediated). However, the recent failure of pegvorhyaluronidase alfa (PEGPH20) combined with chemotherapy in patients with high HA (HA^high^) metastatic pancreatic adenocarcinoma warrants caution. PEGPH20, a PE-Gylated recombinant human hyaluronidase, was developed to enhance the delivery of systemic therapies by degrading HA and TME remodeling.^187–189^ Despite a strong mechanistic rationale, the phase 3 HALO-109–301^190^ trial revealed increased adverse effects and no survival benefit in pancreatic cancer, underscoring the need for rigorous preclinical and clinical evaluation of CAF-targeting strategies. New data suggest that excessive bile acids activate GPBAR1 on CAFs and upregulate CXCL10 increasing invasiveness of iCCA cells, which can create an immunosuppressive TME by recruiting neutrophils.^191^ In animal models, targeting GPBAR1 with specific inhibitors or CXCL10 reduces CCA immunosuppression and boosts anti-PD-1 efficacy,^191^ thus opening new therapeutic avenues. Although still in the early stages, CAF-based therapies are gaining momentum as new therapeutic targets in iCCA and other tumors like pancreatic cancer, which exhibit strong stromal reactions. Similar to immune-based therapies, targeting other components of the cancer ecosystem offers an orthogonal mechanism that complements tumor-directed therapies, potentially maximizing cancer response.

## LIVER CANCER BREAKTHROUGHS FOR THE NEXT DECADE

Artificial intelligence (AI) is poised to transform healthcare by providing tools that enhance our ability to target cancer hallmarks and improve patient outcomes. Machine learning and deep learning models have been applied to a range of clinical challenges in liver cancer, including risk stratification, early detection, outcome prediction, and treatment response.^192^ A recent study that combined AI-based radiomic and clinical data achieved an impressive area under the curve (AUC) of 0.75–0.89 in predicting survival for patients with advanced HCC treated with atezolizumab plus bevacizumab.^104^ Another larger study applied AI to histological H&E slides, developing a model that accurately stratified patients based on progression-free survival.^193^ Additionally, a transformer-based deep learning model, using digitized H&E slides from 431 resected HCC patients, effectively predicted molecular classes and the presence of vascular invasion.^194^ Similar AI methodologies trained on histological features have also been successful in accurately predicting survival outcomes in patients with iCCA.^195^

### Future therapies in HCC

The pace of change in medicine is accelerating. Since the first Hallmarks of Cancer,^6^ HCC has advanced from no effective therapies in 2000 to about 10 targeted therapies or immunotherapies today, and immunotherapy has expanded treatment options and improved survival outcomes. What lies ahead? We anticipate treatment breakthroughs for liver cancer to fall into two main categories. For advanced tumors showing clinical benefit, targeting additional checkpoints (e.g., triple combinations such as atezolizumab-bevacizumab plus anti-TIGIT [IMbrave152; NCT05904886] or tremelimumab-bevacizumab plus rilvegostomig, a bispecific antibody simultaneously targeting PD-1 and TIGIT [ARTEMIDE-HCC; NCT06921785]) is likely to enhance antitumoral activity. However, the toxicity associated with triple or quadruple treatment regimens could be a significant limiting factor. At this point, biomarkers are essential to avoid unnecessary toxicities and improve cost- efficiency. Vaccines and CAR-T therapies, to engage other aspects of the immune response, may further enhance immune responses in these patients.

For patients who exhibit primary resistance to immune-based therapies, treatment strategies will focus on exploiting emerging cancer hallmarks or at overcoming the immunosuppressive TME through drugs inhibiting Wnt/β-catenin^65^ or TGF-β signaling.^196^

The tumor microbiome is now recognized as a dynamic modulator of immune responses, drug metabolism, and tumor progression, including data in HCC.^197^ Microbial signatures could serve as predictive biomarkers or even therapeutic targets, with microbiota engineering offering a novel axis of intervention. Cancer stemness, characterized by self-renewal and therapy resistance, is another frontier.

Stem-like tumor cells and dormancy pathways^198^ contribute to cancer relapse and metastasis, and their unique transcriptional and epigenetic profiles may guide the development of stemness-targeted therapies. Traditionally considered “undruggable” oncogenes, such as *TERT*, *TP53*, *CTNNB1*, *MYC*, and *RAS*, may soon become accessible for selective intervention. Non-canonical components of the TME such as neural innervation^199^ or platelets^200^ are also gaining attention. For patients with early disease (adjuvant/neoadjuvant setting) or intermediate-stage disease, advances such as ICI combined with TACE^84,85^ are expected to significantly impact the management of these patients. We believe the coming decades will usher in more refined tools to match each patient with the most effective therapy, sparing patients from unnecessary toxicity. Ultimately, the convergence of biology, technology, and data science holds promise for introducing a new dimension to understanding cancer hallmarks and transforming the treatment approach for liver cancer.

### Future therapies in iCCA

With the clinical uptake of biomarker-selected therapies for iCCA, comprehensive molecular profiling has emerged as a standard practice.^201^ In the future, the expansion of molecular profiling with more sensitive sequencing technologies, both tissue- and blood-based, may identify a greater proportion of patients eligible for existing targeted therapies, as well as increasing enrollment to biomarker-selected clinical trials. The expanding repertoire of therapeutic targets, however, also accentuates challenges by subdividing the disease into even rarer molecular subgroups, resulting in an inverse impact on feasibility. There, we envision that further progress (Table 2) will require parallel implementation of pragmatic clinical trial designs to enhance efficiency and capture a greater proportion of iCCA patients. Such designs may include master protocols with a shared, concurrent control arm, collaboration with advocacy communities, as well as the use of AI for patient identification and decentralization. In the next decade, we expect an increasing number of research efforts in identifying and validating biomarkers of response to the current standard of care for better patient stratification. Similarly, we anticipate increasing endeavors aimed at identifying alternative ICI-based strategies for patients progressing or primary resistant to the standard of care. In this regard, the application of single-cell-based sequencing paired with spatial technologies and AI in patient samples before and on treatment holds promise to identify the new generation of predictive and prognostic biomarkers. These efforts will certainly require more systematically integrated biospecimen collection to inform the next generation of target identification and trial development. Furthermore, these efforts should be paralleled by the development of more sophisticated patient-derived platforms able to closely recapitulate the hallmarks of the primary tumor and its surrounding microenvironment, as well as their interactions, which can be used in large-scale drug screenings that can inform clinical decisions in real time.

## Figures and Tables

**Table 1:** Recurrent genomic alterations in HCC based on deep-sequencing analyses

Pathway	Gene	Prevalence	Cancer hallmark
**Mutations**			
Telomere stability	*TERT* promoter	432/774 (56%)	enabling replicative immortality
Cell-cycle control	*TP53*	344/1,122 (31%)	genome instability and mutation, evading growth suppressors
	*CDKN2A*	16/1,034 (1%)	evading growth suppressors
	*ATM*	33/1,122 (3%)	genome instability and mutation
	*RB1*	37/1,122 (3%)	evading growth suppressors
Wnt/β-catenin signaling	*CTNNB1*	281/1,122 (25%)	invasion and metastasis
	*AXIN1*	67/1,122 (6%)	
	*APC*	20/1,122 (2%)	
Chromatin remodeling	*ARID1A*	74/1,122 (7%)	genome instability and mutation, non-mutational epigenetic reprogramming
	*ARID2*	69/1,122 (6%)	
	*KMT2A*	30/1,034 (3%)	
	*KMT2C*	39/1,122 (3%)	
	*KMT2B*	17/1,034 (2%)	
Ras/PI3K/mTOR pathway	*RPS6KA3*	39/1,122 (4%)	sustaining proliferative signaling
	*PTEN*	15/1,122 (1%)	
	*PIK3CA*	19/1,122 (2%)	
	*RAS* ^a^	16/1,122 (1%)	
Oxidative stress	*NFE2L2*	33/1,034 (3%)	deregulating metabolism, induction of oxidative stress
	*KEAP1*	35/1,122 (3%)	
JAK/STAT signaling	*JAK1*	24/1,122 (2%)	sustaining proliferative signaling
PDGFR signaling	*PDGFRA*	10/1,016 (1%)	
IGF signaling	*IGF2R*	14/1,034 (1%)	
**High-level focal amplifications**			
VEGF signaling	*VEGFA*	17/547 (3%)	inducing or accessing vasculature
FGF signaling	*FGF19*	37/643 (6%)	sustaining proliferative signaling
Cell-cycle control	*CCND1*	47/643 (7%)	sustaining proliferative signaling
TERT signaling	*TERT*	10/547 (2%)	enabling replicative immortality
**Homozygous deletions**			
Cell-cycle control	*CDKN2A*	28/554 (5%)	evading growth suppressors
	*TP53*	21/554 (4%)	evading growth suppressors, genome instability and mutation
	*RB1*	21/554 (4%)	evading growth suppressors, sustaining proliferative signaling
Wnt/β-catenin signaling	*AXIN1*	16/547 (3%)	sustaining proliferative signaling

a Updated from Llovet et al.^20^

**Table 2:** Current and emerging molecular targets in intrahepatic cholangiocarcinoma

Molecular target	Type of alterations (149, 152, 182–185, 193, 208, 250)	Drug	FDA status
FGFR2	gene fusions (89/587, 15%); mutations (17/480, 3.5%); extracellular domain in-frame deletions (5/178, 2.8%)	pemigatinib	accelerated approval^a^
	gene fusions (89/587, 15%); mutations (17/480, 3.5%); extracellular domain in-frame deletions (5/178, 2.8%)	futibatinib	accelerated approval^a^
	gene fusions (89/587, 15%); mutations (17/480, 3.5%); extracellular domain in-frame deletions (5/178, 2.8%)	lirafugratinib (RLY-4008)	in clinical trial
	gene fusions (89/587, 15%); mutations (17/480, 3.5%); extracellular domain in-frame deletions (5/178, 2.8%)	tinengotinib (TT-00420)	in clinical trial
	gene fusions (89/587, 15%); mutations (17/480, 3.5%); extracellular domain in-frame deletions (5/178, 2.8%)	derazatinib (ARQ 087)	in clinical trial
	gene fusions (89/587, 15%); mutations (17/480, 3.5%); extracellular domain in-frame deletions (5/178, 2.8%)	pemigatinib + afatinib	in clinical trial
IDH1	mutations (52/480, 11%)	ivosidenib	approved^b^
NTRK	gene fusions (2%–3%)	larotrectinib	approved^b^
dMMR and/ or MSI	0.5%–6%	pembrolizumab	approved^b^
BRAF	mutation V600E (11/480, 2%)	dabrafenib plus trametinib	approved^b^
ERBB2	mutations (1%–2%); amplification/ overexpression	zanidatamab	accelerated approval^a^
	mutations (1%–2%); amplification/ overexpression	trastuzumab deruxtecan (Enhertu, T-DXd)	tumor-agnostic accelerated approval^a^
KRAS	mutation G12C (<5%)	adagrasib	not approved
	mutations (82/480,17%)	RMC-6236	in clinical trial
B7-H4	overexpression (40%)	antibody-drug conjugates (AZD8205, SGN-B7H4V, GSK5733584)	in clinical trial

a FDA-approved.

b FDA- and EMA-approved.

## References

[1] Bray F, Laversanne M, Sung H, Ferlay J, Siegel RL, Soerjomataram I, and Jemal A (2024). Global cancer statistics 2022: GLOBOCAN estimates of incidence and mortality worldwide for 36 cancers in 185 countries. CA Cancer J. Clin. 74, 229–263. 10.3322/caac.21834. 38572751

[2] Chan SL, Sun HC, Xu Y, Zeng H, El-Serag HB, Lee JM, Schwartz ME, Finn RS, Seong J, Wang XW, (2025). The Lancet Commission on addressing the global hepatocellular carcinoma burden: comprehensive strategies from prevention to treatment. Lancet 406, 731–778. 10.1016/S0140-6736(25)01042-6. 40744051

[3] Llovet JM, Kelley RK, Villanueva A, Singal AG, Pikarsky E, Roayaie S, Lencioni R, Koike K, Zucman-Rossi J, and Finn RS (2021). Hepatocellular carcinoma. Nat. Rev. Dis. Primers 7, 6. 10.1038/s41572-020-00240-3. 33479224 PMC13537433

[4] Brindley PJ, Bachini M, Ilyas SI, Khan SA, Loukas A, Sirica AE, Teh BT, Wongkham S, and Gores GJ (2021). Cholangiocarcinoma. Nat. Rev. Dis. Primers 7, 65. 10.1038/s41572-021-00300-2. 34504109 PMC9246479

[5] Schulze K, Imbeaud S, Letouzé E, Alexandrov LBLB, Calderaro J, Rebouissou S, Couchy G, Meiller C, Shinde J, Soysouvanh F, (2015). Exome sequencing of hepatocellular carcinomas identifies new mutational signatures and potential therapeutic targets. Nat. Genet. 47, 505–511. 10.1038/ng.3252. 25822088 PMC4587544

[6] Hanahan D, and Weinberg RA (2000). The hallmarks of cancer. Cell 100, 57–70. 10.1016/s0092-8674(00)81683-9. 10647931

[7] Hanahan D, and Weinberg RA (2011). Hallmarks of cancer: the next generation. Cell 144, 646–674. 10.1016/j.cell.2011.02.013. 21376230

[8] Hanahan D (2026). Hallmarks of cancer-Then and now, and beyond. Cell. 10.1016/j.cell.2025.12.049. 41616779

[9] Rumgay H, Arnold M, Ferlay J, Lesi O, Cabasag CJ, Vignat J, Laversanne M, McGlynn KA, and Soerjomataram I (2022). Global burden of primary liver cancer in 2020 and predictions to 2040. J. Hepatol. 77, 1598–1606. 10.1016/j.jhep.2022.08.021. 36208844 PMC9670241

[10] Pinyol R, Torrecilla S, Wang H, Montironi C, Piqué-Gili M, Torres-Martin M, Wei-Qiang L, Willoughby CE, Ramadori P, Andreu-Oller C, (2021). Molecular characterisation of hepatocellular carcinoma in patients with non-alcoholic steatohepatitis. J. Hepatol. 75, 865–878. 10.1016/j.jhep.2021.04.049. 33992698 PMC12164395

[11] Llovet JM, Willoughby CE, Singal AG, Greten TF, Heikenwälder M, El-Serag HB, Finn RS, and Friedman SL (2023). Nonalcoholic steatohepatitis-related hepatocellular carcinoma: pathogenesis and treatment. Nat. Rev. Gastroenterol. Hepatol. 20, 487–503. 10.1038/s41575-023-00754-7. 36932227 PMC12165718

[12] Mauro E, de Castro T, Zeitlhoefler M, Sung MW, Villanueva A, Mazzaferro V, and Llovet JM (2025). Hepatocellular Carcinoma: Epidemiology, diagnosis and treatment. JHEP Rep. 7, 101571. 10.1016/j.jhepr.2025.101571. 41244300 PMC12615749

[13] Llovet JM, Pinyol R, Kelley RK, El-Khoueiry A, Reeves HL, Wang XW, Gores GJ, and Villanueva A (2022). Molecular pathogenesis and systemic therapies for hepatocellular carcinoma. Nat. Cancer 3, 386–401. 10.1038/S43018-022-00357-2. 35484418 PMC9060366

[14] Zucman-Rossi J, Villanueva A, Nault JC, and Llovet JM (2015). Genetic Landscape and Biomarkers of Hepatocellular Carcinoma. Gastroenterology 149, 1226–1239.e4. 10.1053/j.gastro.2015.05.061. 26099527

[15] Michalopoulos GK, and Bhushan B (2021). Liver regeneration: biological and pathological mechanisms and implications. Nat. Rev. Gastroenterol. Hepatol. 18, 40–55. 10.1038/s41575-020-0342-4. 32764740

[16] Sia D, Villanueva A, Friedman SL, and Llovet JM (2017). Liver Cancer Cell of Origin, Molecular Class, and Effects on Patient Prognosis. Gastroenterology 152, 745–761. 10.1053/j.gastro.2016.11.048. 28043904 PMC12160040

[17] Moeini A, Torrecilla S, Tovar V, Montironi C, Andreu-Oller C, Peix J, Higuera M, Pfister D, Ramadori P, Pinyol R, (2019). An Immune Gene Expression Signature Associated With Development of Human Hepatocellular Carcinoma Identifies Mice That Respond to Chemopreventive Agents. Gastroenterology 157, 1383–1397.e11. 10.1053/j.gastro.2019.07.028. 31344396 PMC6815707

[18] Guo J, Liang R, Chung A, Li Z, Li B, Chen E, Li L, Wang J, Hsieh MH, Fang IX, (2026). The origin of hepatocellular carcinoma depends on metabolic zonation. Science 391, eadv7129. 10.1126/science.adv7129. 41196951 PMC12999168

[19] Raven A, Gilroy K, Jin H, Waldron JA, Leslie H, Munro J, Hall H, Ridgway RA, Ford CA, Gulhan DC, (2026). Hepatic zonation determines tumorigenic potential of mutant β-catenin. Nature 649, 739–748. 10.1038/s41586-025-09733-1. 41261129 PMC12804091

[20] Llovet JM, Villanueva A, Lachenmayer A, and Finn RS (2015). Advances in targeted therapies for hepatocellular carcinoma in the genomic era. Nat. Rev. Clin. Oncol. 12, 408–424. 10.1038/nrcli-nonc.2015.103. 26054909

[21] Villanueva A (2019). Hepatocellular Carcinoma. N. Engl. J. Med. 380, 1450–1462. 10.1056/NEJMra1713263. 30970190

[22] Ningarhari M, Caruso S, Hirsch TZ, Bayard Q, Franconi A, Védie A-L, Noblet B, Blanc J-F, Amaddeo G, Ganne N, (2021). Telomere length is key to hepatocellular carcinoma diversity and telomerase addiction is an actionable therapeutic target. J. Hepatol. 74, 1155–1166. 10.1016/j.jhep.2020.11.052. 33338512

[23] Pinyol R, Tovar V, and Llovet JMJM (2014). TERT promoter mutations: Gatekeeper and driver of hepatocellular carcinoma. J. Hepatol. 61, 685–687. 10.1016/j.jhep.2014.05.028. 24859456

[24] Zoulim F, Chen PJ, Dandri M, Kennedy PT, and Seeger C (2024). Hepatitis B virus DNA integration: Implications for diagnostics, therapy, and outcome. J. Hepatol. 81, 1087–1099. 10.1016/j.jhep.2024.06.037. 38971531

[25] Kastenhuber ER, and Lowe SW (2017). Putting p53 in context. Cell 170, 1062–1078. 10.1016/j.cell.2017.08.028. 28886379 PMC5743327

[26] Sun J, and Cheng NS (2022). Comprehensive Landscape of ARID Family Members and Their Association with Prognosis and Tumor Microenvironment in Hepatocellular Carcinoma. J. Immunol. Res. 2022, 1688460. 10.1155/2022/1688460. 35402625 PMC8986425

[27] Chiang DY, Villanueva A, Hoshida Y, Peix J, Newell P, Minguez B, LeBlanc AC, Donovan DJ, Thung SN, Solé M, (2008). Focal Gains of VEGFA and Molecular Classification of Hepatocellular Carcinoma. Cancer Res. 68, 6779–6788. 10.1158/0008-5472.CAN-08-0742. 18701503 PMC2587454

[28] Davoli T, Uno H, Wooten EC, and Elledge SJ (2017). Tumor aneuploidy correlates with markers of immune evasion and with reduced response to immunotherapy. Science 355, eaaf8399. 10.1126/science.aaf8399. 28104840 PMC5592794

[29] Bassaganyas L, Pinyol R, Esteban-Fabró R, Torrens L, Torrecilla S, Willoughby CE, Franch-Expósito S, Vila-Casadesús M, Salaverria I, Montal R, (2020). Copy-Number Alteration Burden Differentially Impacts Immune Profiles and Molecular Features of Hepatocellular Carcinoma. Clin. Cancer Res. 26, 6350–6361. 10.1158/1078-0432.CCR-20-1497. 32873569 PMC7710584

[30] Fitamant J, Kottakis F, Benhamouche S, Tian HS, Chuvin N, Parachoniak CA, Nagle JM, Perera RM, Lapouge M, Deshpande V, (2015). YAP Inhibition Restores Hepatocyte Differentiation in Advanced HCC, Leading to Tumor Regression. Cell Rep. 10, 1692–1707. 10.1016/j.celrep.2015.02.027. 25772357 PMC4565791

[31] Zender L, Spector MS, Xue W, Flemming P, Cordon-Cardo C, Silke J, Fan ST, Luk JM, Wigler M, Hannon GJ, (2006). Identification and validation of oncogenes in liver cancer using an integrative oncogenomic approach. Cell 125, 1253–1267. 10.1016/j.cell.2006.05.030. 16814713 PMC3026384

[32] Xue W, Kitzing T, Roessler S, Zuber J, Krasnitz A, Schultz N, Revill K, Weissmueller S, Rappaport AR, Simon J, (2012). A cluster of cooperating tumor-suppressor gene candidates in chromosomal deletions. Proc. Natl. Acad. Sci. USA 109, 8212–8217. 10.1073/pnas.1206062109. 22566646 PMC3361457

[33] Liu Y, Chen C, Xu Z, Scuoppo C, Rillahan CD, Gao J, Spitzer B, Bosbach B, Kastenhuber ER, Baslan T, (2016). Deletions linked to TP53 loss drive cancer through p53-independent mechanisms. Nature 531, 471–475. 10.1038/nature17157. 26982726 PMC4836395

[34] Klocke R, Bartels T, Jennings G, Brand K, Halter R, Strauss M, and Paul D (2001). Lack of p53 accelerates hepatocarcinogenesis in transgenic mice constitutively overexpressing c-myc in the liver. FASEB J. 15, 1404–1406. 10.1096/fj.00-0487fje. 11387238

[35] Zhu C, Soto-Feliciano YM, Morris JP, Huang CH, Koche RP, Ho YJ, Banito A, Chen CW, Shroff A, Tian S, (2023). MLL3 regulates the *CDKN2A* tumor suppressor locus in liver cancer. eLife 12, e80854. 10.7554/eLife.80854. 37261974 PMC10279454

[36] Calvisi DF, Ladu S, Gorden A, Farina M, Lee JS, Conner EA, Schroeder I, Factor VM, and Thorgeirsson SS (2007). Mechanistic and prognostic significance of aberrant methylation in the molecular pathogenesis of human hepatocellular carcinoma. J. Clin. Investig. 117, 2713–2722. 10.1172/JCI31457. 17717605 PMC1950459

[37] Guichard C, Amaddeo G, Imbeaud S, Ladeiro Y, Pelletier L, Maad IB, Calderaro J, Bioulac-Sage P, Letexier M, Degos F, (2012). Integrated analysis of somatic mutations and focal copy-number changes identifies key genes and pathways in hepatocellular carcinoma. Nat. Genet. 44, 694–698. 10.1038/ng.2256. 22561517 PMC3819251

[38] Weissmueller S, Manchado E, Saborowski M, Morris JP, Wagenblast E, Davis CA, Moon SH, Pfister NT, Tschaharganeh DF, Kitzing T, (2014). Mutant p53 drives pancreatic cancer metastasis through cell-autonomous PDGF receptor β signaling. Cell 157, 382–394. 10.1016/j.cell.2014.01.066. 24725405 PMC4001090

[39] Revia S, Seretny A, Wendler L, Banito A, Eckert C, Breuer K, Mayakonda A, Lutsik P, Evert M, Ribback S, (2022). Histone H3K27 demethylase KDM6A is an epigenetic gatekeeper of mTORC1 signalling in cancer. Gut 71, 1613–1628. 10.1136/gutjnl-2021-325405. 34509979 PMC9279849

[40] Bueloni B, G Fernandez-Barrena M, Fiore E, Avila MA, Bayo J, and Mazzolini GD (2025). Epigenetic therapies in hepatocellular carcinoma: emerging clinical tools and applications. Gut. 10.1136/gutjnl-2025-336317. 40987534

[41] Müller M, May S, Hall H, Kendall TJ, McGarry L, Blukacz L, Nuciforo S, Georgakopoulou A, Jamieson T, Phinichkusolchit N, (2025). Human-correlated genetic models identify precision therapy for liver cancer. Nature 639, 754–764. 10.1038/s41586-025-08585-z. 39972137 PMC11922762

[42] Sia D, Jiao Y, Martinez-Quetglas I, Kuchuk O, Villacorta-Martin C, Castro de Moura M, Putra J, Camprecios G, Bassaganyas L, Akers N, (2017). Identification of an Immune-specific Class of Hepatocellular Carcinoma, Based on Molecular Features. Gastroenterology 153, 812–826. 10.1053/j.gastro.2017.06.007. 28624577 PMC12166766

[43] Montironi C, Castet F, Haber PK, Pinyol R, Torres-Martin M, Torrens L, Mesropian A, Wang H, Puigvehi M, Maeda M, (2023). Inflamed and non-inflamed classes of HCC: a revised immunogenomic classification. Gut 72, 129–140. 10.1136/gutjnl-2021-325918. 35197323 PMC9395551

[44] Llovet JM, Castet F, Heikenwalder M, Maini MK, Mazzaferro V, Pinato DJ, Pikarsky E, Zhu AX, and Finn RS (2022). Immunotherapies for hepatocellular carcinoma. Nat. Rev. Clin. Oncol. 19, 151–172. 10.1038/s41571-021-00573-2. 34764464

[45] Barcena-Varela M, Monga SP, and Lujambio A (2025). Precision models in hepatocellular carcinoma. Nat. Rev. Gastroenterol. Hepatol. 22, 191–205. 10.1038/s41575-024-01024-w. 39663463

[46] Kim RD, Sarker D, Meyer T, Yau T, Macarulla T, Park JW, Choo SP, Hollebecque A, Sung MW, Lim HY, (2019). First-in-Human Phase I Study of Fisogatinib (BLU-554) Validates Aberrant FGF19 Signaling as a Driver Event in Hepatocellular Carcinoma. Cancer Discov. 9, 1696–1707. 10.1158/2159-8290.CD-19-0555. 31575541

[47] Chan SL, Schuler M, Kang YK, Yen CJ, Edeline J, Choo SP, Lin CC, Okusaka T, Weiss KH, Macarulla T, (2022). A first-in-human phase 1/2 study of FGF401 and combination of FGF401 with spartalizumab in patients with hepatocellular carcinoma or biomarker-selected solid tumors. J. Exp. Clin. Cancer Res. 41, 189. 10.1186/s13046-022-02383-5. 35655320 PMC9161616

[48] Bollard J, Miguela V, Ruiz de Galarreta M, Venkatesh A, Bian CB, Roberto MP, Tovar V, Sia D, Molina-Sánchez P, Nguyen CB, (2017). Palbociclib (PD-0332991), a selective CDK4/6 inhibitor, restricts tumour growth in preclinical models of hepatocellular carcinoma. Gut 66, 1286–1296. 10.1136/gutjnl-2016-312268. 27849562 PMC5512174

[49] de Zawadzki A, Leeming DJ, Sanyal AJ, Anstee QM, Schattenberg JM, Friedman SL, Schuppan D, and Karsdal MA (2025). Hot and cold fibrosis: The role of serum biomarkers to assess immune mechanisms and ECM-cell interactions in human fibrosis. J. Hepatol. 83, 239–257. 10.1016/j.jhep.2025.02.039. 40056933

[50] Morse MA, Sun W, Kim R, He AR, Abada PB, Mynderse M, and Finn RS (2019). The Role of Angiogenesis in Hepatocellular Carcinoma. Clin. Cancer Res. 25, 912–920. 10.1158/1078-0432.CCR-18-1254. 30274981

[51] Llovet JM, Ricci S, Mazzaferro V, Hilgard P, Gane E, Blanc J-F, de Oliveira AC, Santoro A, Raoul J-L, Forner A, (2008). Sorafenib in Advanced Hepatocellular Carcinoma. N. Engl. J. Med. 359, 378–390. 10.1056/NEJMoa0708857. 18650514

[52] Wilhelm SM, Adnane L, Newell P, Villanueva A, Llovet JM, and Lynch M (2008). Preclinical overview of sorafenib, a multikinase inhibitor that targets both Raf and VEGF and PDGF receptor tyrosine kinase signaling. Mol. Cancer Ther. 7, 3129–3140. 10.1158/1535-7163.MCT-08-0013. 18852116 PMC12261297

[53] Kudo M, Finn RS, Qin S, Han K-HH, Ikeda K, Piscaglia F, Baron A, Park J-WW, Han G, Jassem J, (2018). Lenvatinib versus sorafenib in first-line treatment of patients with unresectable hepatocellular carcinoma: a randomised phase 3 non-inferiority trial. Lancet 391, 1163–1173. 10.1016/S0140-6736(18)30207-1. 29433850

[54] Bruix J, Qin S, Merle P, Granito A, Huang Y-HH, Bodoky G, Pracht M, Yokosuka O, Rosmorduc O, Breder V, (2017). Regorafenib for patients with hepatocellular carcinoma who progressed on sorafenib treatment (RESORCE): a randomised, double-blind, placebo-controlled, phase 3 trial. Lancet 389, 56–66. 10.1016/S0140-6736(16)32453-9. 27932229

[55] Abou-Alfa GK, Meyer T, Cheng A-L, El-Khoueiry AB, Rimassa L, Ryoo B-Y, Cicin I, Merle P, Chen Y, Park J-W, (2018). Cabozantinib in Patients with Advanced and Progressing Hepatocellular Carcinoma. N. Engl. J. Med. 379, 54–63. 10.1056/NEJMoa1717002. 29972759 PMC7523244

[56] Qin S, Chan SL, Gu S, Bai Y, Ren Z, Lin X, Chen Z, Jia W, Jin Y, Guo Y, (2023). Camrelizumab plus rivoceranib versus sorafenib as first-line therapy for unresectable hepatocellular carcinoma (CARES-310): a randomised, open-label, international phase 3 study. Lancet 402, 1133–1146. 10.1016/S0140-6736(23)00961-3. 37499670

[57] Zhu AX, Kang YK, Yen CJ, Finn RS, Galle PR, Llovet JM, As senat E, Brandi G, Pracht M, Lim HY, (2019). Ramucirumab after sorafenib in patients with advanced hepatocellular carcinoma and increased α-fetoprotein concentrations (REACH-2): a randomised, double-blind, placebo-controlled, phase 3 trial. Lancet Oncol. 20, 282–296. 10.1016/S1470-2045(18)30937-9. 30665869

[58] Xing R, Gao J, Cui Q, and Wang Q (2021). Strategies to Improve the Antitumor Effect of Immunotherapy for Hepatocellular Carcinoma. Front. Immunol. 12, 783236. 10.3389/fimmu.2021.783236. 34899747 PMC8660685

[59] Finn RS, Qin S, Ikeda M, Galle PR, Ducreux M, Kim TY, Kudo M, Breder V, Merle P, Kaseb AO, (2020). Atezolizumab plus Bevacizumab in Unresectable Hepatocellular Carcinoma. N. Engl. J. Med. 382, 1894–1905. 10.1056/NEJMoa1915745. 32402160

[60] Zhu AX, Abbas AR, de Galarreta MR, Guan Y, Lu S, Koeppen H, Zhang W, Hsu CH, He AR, Ryoo BY, (2022). Molecular correlates of clinical response and resistance to atezolizumab in combination with bevacizumab in advanced hepatocellular carcinoma. Nat. Med. 28, 1599–1611. 10.1038/s41591-022-01868-2. 35739268

[61] Lachenmayer A, Alsinet C, Savic R, Cabellos L, Toffanin S, Hoshida Y, Villanueva A, Minguez B, Newell P, Tsai H-W, (2012). Wnt-Pathway Activation in Two Molecular Classes of Hepatocellular Carcinoma and Experimental Modulation by Sorafenib. Clin. Cancer Res. 18, 4997–5007. 10.1158/1078-0432.CCR-11-2322. 22811581 PMC3446854

[62] Pinyol R, Sia D, and Llovet JM (2019). Immune Exclusion-Wnt/CTNNB1 Class Predicts Resistance to Immunotherapies in HCC. Clin. Cancer Res. 25, 2021–2023. 10.1158/1078-0432.CCR-18-3778. 30617138 PMC6445700

[63] Ruiz de Galarreta M, Bresnahan E, Molina-Sánchez P, Lindblad KE, Maier B, Sia D, Puigvehi M, Miguela V, Casanova-Acebes M, Dhainaut M, (2019). β-Catenin Activation Promotes Immune Escape and Resistance to Anti–PD-1 Therapy in Hepatocellular Carcinoma. Cancer Discov. 9, 1124–1141. 10.1158/2159-8290.CD-19-0074. 31186238 PMC6677618

[64] Krishna A, Meynert A, Dolt KS, Kelder M, Mesropian A, Ewing A, Brouwers C, Claassens JW, Linssen MM, Sheraz S, (2026). Mutational scanning reveals oncogenic CTNNB1 mutations have diverse effects on signaling. Nat. Genet. 58, 366–375. 10.1038/s41588-025-02496-5. 41629672 PMC12900645

[65] Rialdi A, Duffy M, Scopton AP, Fonseca F, Zhao JN, Schwarz M, Molina-Sanchez P, Mzoughi S, Arceci E, Abril-Fornaguera J, (2023). WNTinib is a Multi-Kinase Inhibitor with Specificity Against β-Catenin Mutant Hepatocellular Carcinoma (Springer). 10.1038/s43018-023-00609-9. PMC10948969 37537299

[66] Mesropian A, Gris-Oliver A, Balaseviciute U, Potdar AA, Kimura T, Shen J, Torres-Marcén M, Abril-Fornaguera J, Piqué-Gili M, Camell-Raventos D, (2025). E7386 enhances lenvatinib’s antitumor activity in preclinical models and human hepatocellular carcinoma. Clin. Cancer Res. 31, 5037–5050. 10.1158/1078-0432.CCR-25-0725. 40986544 PMC12666316

[67] Nevzorova YA, Hu W, Cubero FJ, Haas U, Freimuth J, Tacke F, Trautwein C, and Liedtke C (2013). Overexpression of c-myc in hepatocytes promotes activation of hepatic stellate cells and facilitates the onset of liver fibrosis. Biochim. Biophys. Acta 1832, 1765–1775. 10.1016/j.bbadis.2013.06.001. 23770341

[68] Müller M, Bird TG, and Nault JC (2020). The landscape of gene mutations in cirrhosis and hepatocellular carcinoma. J. Hepatol. 72, 990–1002. 10.1016/j.jhep.2020.01.019. 32044402

[69] Sanyal AJ, Newsome PN, Kliers I, Østergaard LH, Long MT, Kjær MS, Cali AMG, Bugianesi E, Rinella ME, Roden M, (2025). Phase 3 Trial of Semaglutide in Metabolic Dysfunction-Associated Steatohepatitis. N. Engl. J. Med. 392, 2089–2099. 10.1056/NEJMoa2413258. 40305708

[70] Harrison SA, Bedossa P, Guy CD, Schattenberg JM, Loomba R, Taub R, Labriola D, Moussa SE, Neff GW, Rinella ME, (2024). A Phase 3, Randomized, Controlled Trial of Resmetirom in NASH with Liver Fibrosis. N. Engl. J. Med. 390, 497–509. 10.1056/NEJMoa2309000. 38324483

[71] Chew V, Lai L, Pan L, Lim CJ, Li J, Ong R, Chua C, Leong JY, Lim KH, Toh HC, (2017). Delineation of an immunosuppressive gradient in hepatocellular carcinoma using high-dimensional proteomic and transcriptomic analyses. Proc. Natl. Acad. Sci. USA 114, E5900–E5909. 10.1073/pnas.1706559114. 28674001 PMC5530700

[72] Yarchoan M, Albacker LA, Hopkins AC, Montesion M, Murugesan K, Vithayathil TT, Zaidi N, Azad NS, Laheru DA, Frampton GM, (2019). PD-L1 expression and tumor mutational burden are independent biomarkers in most cancers. JCI Insight 4, e126908. 10.1172/jci.insight.126908. 30895946 PMC6482991

[73] Cappuyns S, Piqué-Gili M, Esteban-Fabró R, Philips G, Balaseviciute U, Pinyol R, Gris-Oliver A, Vandecaveye V, Abril-Fornaguera J, Montironi C, (2025). Single-cell RNA sequencing-derived signatures define response patterns to atezolizumab + bevacizumab in advanced hepatocellular carcinoma. J. Hepatol. 82, 1036–1049. 10.1016/j.jhep.2024.12.016. 39709141 PMC12086051

[74] Lehrich BM, and Monga SP (2025). WNT–β-catenin signalling in hepatocellular carcinoma: from bench to clinical trials. Nat. Rev. Gastroenterol. Hepatol. 23, 246–263. 10.1038/s41575-025-01127-y. 41068417 PMC13560752

[75] Sangro B, Chan SL, Kelley RK, Lau G, Kudo M, Sukeepaisarnjaroen W, Yarchoan M, De Toni EN, Furuse J, Kang YK, (2024). Four-year overall survival update from the phase III HIMALAYA study of tremelimumab plus durvalumab in unresectable hepatocellular carcinoma. Ann. Oncol. 35, 448–457. 10.1016/j.annonc.2024.02.005. 38382875

[76] Yau T, Galle PR, Decaens T, Sangro B, Qin S, da Fonseca LG, Karachiwala H, Blanc JF, Park JW, Gane E, (2025). Nivolumab plus ipilimumab versus lenvatinib or sorafenib as first-line treatment for unresectable hepatocellular carcinoma (CheckMate 9DW): an open-label, randomised, phase 3 trial. Lancet 405, 1851–1864. 10.1016/S0140-6736(25)00403-9. 40349714

[77] Wei SC, Duffy CR, and Allison JP (2018). Fundamental Mechanisms of Immune Checkpoint Blockade Therapy. Cancer Discov. 8, 1069–1086. 10.1158/2159-8290.CD-18-0367. 30115704

[78] Qin S, Chen M, Cheng AL, Kaseb AO, Kudo M, Lee HC, Yopp AC, Zhou J, Wang L, Wen X, (2023). Atezolizumab plus bevacizumab versus active surveillance in patients with resected or ablated high-risk hepatocellular carcinoma (IMbrave050): a randomised, open-label, multicentre, phase 3 trial. Lancet 402, 1835–1847. 10.1016/S0140-6736(23)01796-8. 37871608

[79] Llovet JM, Pinyol R, Yarchoan M, Singal AG, Marron TU, Schwartz M, Pikarsky E, Kudo M, and Finn RS (2024). Adjuvant and neoadjuvant immunotherapies in hepatocellular carcinoma. Nat. Rev. Clin. Oncol. 21, 294–311. 10.1038/s41571-024-00868-0. 38424197 PMC11984461

[80] Ho WJ, Zhu Q, Durham J, Popovic A, Xavier S, Leatherman J, Mohan A, Mo G, Zhang S, Gross N, (2021). Neoadjuvant Cabozantinib and Nivolumab Converts Locally Advanced HCC into Resectable Disease with Enhanced Antitumor Immunity. Nat. Cancer 2, 891–903. 10.1038/s43018-021-00234-4. 34796337 PMC8594857

[81] Marron TU, Fiel MI, Hamon P, Fiaschi N, Kim E, Ward SC, Zhao Z, Kim J, Kennedy P, Gunasekaran G, (2022). Neoadjuvant cemiplimab for resectable hepatocellular carcinoma: a single-arm, open-label, phase 2 trial. Lancet Gastroenterol. Hepatol. 7, 219–229. 10.1016/S2468-1253(21)00385-X. 35065058 PMC9901534

[82] Kaseb AO, Hasanov E, Cao HST, Xiao L, Vauthey J-NN, Lee SS, Yavuz BG, Mohamed YI, Qayyum A, Jindal S, (2022). Perioperative nivolumab monotherapy versus nivolumab plus ipilimumab in resectable hepatocellular carcinoma: a randomised, open-label, phase 2 trial. Lancet Gastroenterol. Hepatol. 7, 208–218. 10.1016/S2468-1253(21)00427-1. 35065057 PMC8840977

[83] Wang Z, Fan J, Zhou S, Sun Y, Liang F, Ji Y, Gu F, Li T, Peng L, Peng T, (2025). Perioperative camrelizumab plus rivoceranib versus surgery alone in patients with resectable hepatocellular carcinoma at intermediate or high risk of recurrence (CARES-009): a randomised phase 2/3 trial. Lancet 406, 2089–2099. 10.1016/S0140-6736(25)01720-9. 41125112

[84] Kudo M, Ren Z, Guo Y, Han G, Lin H, Zheng J, Ogasawara S, Kim JH, Zhao H, Li C, (2025). Transarterial chemoembolisation combined with lenvatinib plus pembrolizumab versus dual placebo for unresectable, non-metastatic hepatocellular carcinoma (LEAP-012): a multicentre, randomised, double-blind, phase 3 study. Lancet 405, 203–215. 10.1016/S0140-6736(24)02575-3. 39798578

[85] Sangro B, Kudo M, Erinjeri JP, Qin S, Ren Z, Chan SL, Arai Y, Heo J, Mai A, Escobar J, (2025). Durvalumab with or without bevacizumab with transarterial chemoembolisation in hepatocellular carcinoma (EMERALD-1): a multiregional, randomised, double-blind, placebo-controlled, phase 3 study. Lancet 405, 216–232. 10.1016/S0140-6736(24)02551-0. 39798579 PMC12282661

[86] Rimassa L, Chan SL, Sangro B, Lau G, Kudo M, Reig M, Breder V, Ryu MH, Ostapenko Y, Sukeepaisarnjaroen W, (2025). Five-year overall survival update from the HIMALAYA study of tremelimumab plus durvalumab in unresectable HCC. J. Hepatol. 83, 899–908. 10.1016/j.jhep.2025.03.033. 40222621

[87] Badhrinarayanan S, Cotter C, Zhu H, Lin YC, Kudo M, and Li D (2024). IMbrave152/SKYSCRAPER-14: a Phase III study of atezolizumab, bevacizumab and tiragolumab in advanced hepatocellular carcinoma. Future Oncol. 20, 2049–2057. 10.1080/14796694.2024.2355863. 38861301 PMC11497967

[88] ClinicalTrials.gov (2025). Phase III Study of Rilvegostomig in Combination With Bevacizumab With or Without Tremelimumab as First-line Treatment of Hepatocellular Carcinoma (ARTEMIDE-HCC01). https://clinicaltrials.gov/study/NCT06921785.

[89] Abou-Alfa GK, Bouattour M, Cheng A-L, Dayyani F, Khalil D, Li D, Liao C-Y, López López CL, Rimassa L, Deutsch J, (2024). Phase 2/3 study of livmoniplimab in combination with budigalimab in patients with locally advanced or metastatic hepatocellular carcinoma. J. Clin. Oncol. 42. TPS4190–TPS4190. 10.1200/JCO.2024.42.16_suppl.TPS4190.

[90] Yarchoan M, Gane EJ, Marron TU, Perales-Linares R, Yan J, Cooch N, Shu DH, Fertig EJ, Kagohara LT, Bartha G, (2024). Personalized neoantigen vaccine and pembrolizumab in advanced hepatocellular carcinoma: a phase 1/2 trial. Nat. Med. 30, 1044–1053. 10.1038/s41591-024-02894-y. 38584166 PMC11031401

[91] Yopp A, Kudo M, Chen M, Cheng A-L, Kaseb AO, Lee HC, Qin S, Cha E, Hack SP, Lian Q, (2024). LBA39 Updated efficacy and safety data from IMbrave050: Phase III study of adjuvant atezolizumab (atezo) + bevacizumab (bev) vs active surveillance in patients (pts) with resected or ablated high-risk hepatocellular carcinoma (HCC). Ann. Oncol. 35, S1230. 10.1016/j.annonc.2024.08.2279.

[92] Zaidi N, Jaffee EM, and Yarchoan M (2025). Recent advances in therapeutic cancer vaccines. Nat. Rev. Cancer 25, 517–533. 10.1038/s41568-025-00820-z. 40379970

[93] Meyer T, Finn RS, Borad M, Mahipal A, Edeline J, Houot R, Hausner PF, Hollebecque A, Goyal L, Frigault M, (2025). Phase I trial of ADP-A2AFP TCR T-cell therapy in patients with advanced hepatocellular or gastric hepatoid carcinoma. J. Hepatol. 84, 113–121. 10.1016/j.jhep.2025.07.033. 40812667

[94] Zhang Q, Fu Q, Cao W, Wang H, Xu X, Huang J, Zou A, Zhu J, Wan H, Yao Y, (2024). Phase I study of C-CAR031, a GPC3-specific TGFβRIIDN armored autologous CAR-T, in patients with advanced hepatocellular carcinoma (HCC). J. Clin. Oncol. 42, 4019. 10.1200/JCO.2024.42.16_suppl.4019.

[95] Shen Y, Bai X, Zhang Q, Liang X, Jin X, Zhao Z, Song W, Tan Q, Zhao R, Jia W, (2025). Oncolytic virus VG161 in refractory hepatocellular carcinoma. Nature 641, 503–511. 10.1038/s41586-025-08717-5. 40108464

[96] Heo J, Reid T, Ruo L, Breitbach CJ, Rose S, Bloomston M, Cho M, Lim HY, Chung HC, Kim CW, (2013). Randomized dose-finding clinical trial of oncolytic immunotherapeutic vaccinia JX-594 in liver cancer. Nat. Med. 19, 329–336. 10.1038/nm.3089. 23396206 PMC4268543

[97] Singal AG, Llovet JM, Yarchoan M, Mehta N, Heimbach JK, Dawson LA, Jou JH, Kulik LM, Agopian VG, Marrero JA, (2023). AASLD Practice Guidance on prevention, diagnosis, and treatment of hepatocellular carcinoma. Hepatology 78, 1922–1965. 10.1097/HEP.0000000000000466. 37199193 PMC10663390

[98] Bruix J, Cheng AL, Meinhardt G, Nakajima K, De Sanctis Y, and Llovet J (2017). Prognostic factors and predictors of sorafenib benefit in patients with hepatocellular carcinoma: Analysis of two phase III studies. J. Hepatol. 67, 999–1008. 10.1016/j.jhep.2017.06.026. 28687477

[99] Teufel M, Seidel H, Köchert K, Meinhardt G, Finn RS, Llovet JM, and Bruix J (2019). Biomarkers Associated With Response to Regorafenib in Patients With Hepatocellular Carcinoma. Gastroenterology 156, 1731–1741. 10.1053/j.gastro.2019.01.261. 30738047

[100] Sangro B, Melero I, Wadhawan S, Finn RS, Abou-Alfa GK, Cheng AL, Yau T, Furuse J, Park JW, Boyd Z, (2020). Association of inflammatory biomarkers with clinical outcomes in nivolumab-treated patients with advanced hepatocellular carcinoma. J. Hepatol. 73, 1460–1469. 10.1016/j.jhep.2020.07.026. 32710922 PMC7751218

[101] Haber PK, Castet F, Torres-Martin M, Andreu-Oller C, Puigvehí M, Miho M, Radu P, Dufour J-F, Verslype C, Zimpel C, (2023). Molecular Markers of Response to Anti-PD1 Therapy in Advanced Hepatocellular Carcinoma. Gastroenterology 164, 72–88.e18. 10.1053/j.gastro.2022.09.005. 36108710 PMC12182972

[102] American College of Radiology (2018). Liver Imaging Reporting and Data System (LI-RADS^®^) v2018 Core (American College of Radiology). https://www.acr.org/Clinical-Resources/Reporting-and-Data-Systems/LI-RADS.

[103] von Felden J, Craig AJ, Garcia-Lezana T, Labgaa I, Haber PK, D’Avola D, Asgharpour A, Dieterich D, Bonaccorso A, Torres-Martin M, (2021). Mutations in circulating tumor DNA predict primary resistance to systemic therapies in advanced hepatocellular carcinoma. Oncogene 40, 140–151. 10.1038/s41388-020-01519-1. 33097857 PMC12452111

[104] Vithayathil M, Koku D, Campani C, Nault JC, Sutter O, Ganne-Carrié N, Aboagye EO, and Sharma R (2025). Machine learning based radiomic models outperform clinical biomarkers in predicting outcomes after immunotherapy for hepatocellular carcinoma. J. Hepatol. 83, 959–970. 10.1016/j.jhep.2025.04.017. 40246150

[105] Lewis S, Cedillo MA, Lee KM, Bane O, Hectors S, Ma W, Wang P, Stocker D, Morris DV, Pinato D, (2022). Comparative assessment of standard and immune response criteria for evaluation of response to PD-1 monotherapy in unresectable HCC. Abdom. Radiol. 47, 969–980. 10.1007/s00261-021-03386-0. 34964909

[106] Llovet JM (2023). Exploring a new pathway for biomarker-based approval of immunotherapies. Nat. Rev. Clin. Oncol. 20, 279–280. 10.1038/s41571-023-00731-8. 36707728

[107] D’Alessio A, Stefanini B, Blanter J, Adegbite B, Crowley F, Yip V, Slater S, Fulgenzi CAM, Celsa C, Manfredi GF, (2024). Pathological response following neoadjuvant immune checkpoint inhibitors in patients with hepatocellular carcinoma: a cross-trial, patient-level analysis. Lancet Oncol. 25, 1465–1475. 10.1016/S1470-2045(24)00457-1. 39437804 PMC12040480

[108] O’Dwyer PJ, Gray RJ, Flaherty KT, Chen AP, Li S, Wang V, McShane LM, Patton DR, Tricoli JV, Williams PM, (2023). The NCI-MATCH trial: lessons for precision oncology. Nat. Med. 29, 1349–1357. 10.1038/s41591-023-02379-4. 37322121 PMC10612141

[109] Llovet JM, De Baere T, Kulik L, Haber PK, Greten TF, Meyer T, and Lencioni R (2021). Locoregional therapies in the era of molecular and immune treatments for hepatocellular carcinoma. Nat. Rev. Gastroenterol. Hepatol. 18, 293–313. 10.1038/s41575-020-00395-0. 33510460

[110] Vicent S, Lieshout R, Saborowski A, Verstegen MMA, Raggi C, Recalcati S, Invernizzi P, van der Laan LJW, Alvaro D, Calvisi DF, (2019). Experimental models to unravel the molecular pathogenesis, cell of origin and stem cell properties of cholangiocarcinoma. Liver Int. 39, 79–97. 10.1111/liv.14094. 30851232

[111] Fan B, Malato Y, Calvisi DF, Naqvi S, Razumilava N, Ribback S, Gores GJ, Dombrowski F, Evert M, Chen X, (2012). Cholangiocarcinomas can originate from hepatocytes in mice. J. Clin. Investig. 122, 2911–2915. 10.1172/JCI63212. 22797301 PMC3408746

[112] Hsu BY, Driscoll J, Human; Cholangiocarcinogenesis Project, Tateno C, Mattis AN, Kelley RK, and Willenbring H (2024). Human Hepatocytes Can Give Rise to Intrahepatic Cholangiocarcinomas. Gastroenterology 167, 1029–1032.e7. 10.1053/j.gastro.2024.05.033. 38866344 PMC11753062

[113] European Association for the Study of the Liver (2023). EASL-ILCA Clinical Practice Guidelines on the management of intrahepatic cholangiocarcinoma. J. Hepatol. 79, 181–208. 10.1016/j.jhep.2023.03.010. 37084797

[114] Rumgay H, Ferlay J, de Martel C, Georges D, Ibrahim AS, Zheng R, Wei W, Lemmens VEPP, and Soerjomataram I (2022). Global, regional and national burden of primary liver cancer by subtype. Eur. J. Cancer 161, 108–118. 10.1016/j.ejca.2021.11.023. 34942552

[115] Javle M, Lee S, Azad NS, Borad MJ, Kate Kelley R, Sivaraman S, Teschemaker A, Chopra I, Janjan N, Parasuraman S, (2022). Temporal Changes in Cholangiocarcinoma Incidence and Mortality in the United States from 2001 to 2017. Oncologist 27, 874–883. 10.1093/oncolo/oyac150. 35972334 PMC9526482

[116] Bertuccio P, Malvezzi M, Carioli G, Hashim D, Boffetta P, El-Serag HB, La Vecchia C, and Negri E (2019). Global trends in mortality from intrahepatic and extrahepatic cholangiocarcinoma. J. Hepatol. 71, 104–114. 10.1016/j.jhep.2019.03.013. 30910538

[117] Banales JM, Marin JJG, Lamarca A, Rodrigues PM, Khan SA, Roberts LR, Cardinale V, Carpino G, Andersen JB, Braconi C, (2020). Cholangiocarcinoma 2020: the next horizon in mechanisms and management. Nat. Rev. Gastroenterol. Hepatol. 17, 557–588. 10.1038/s41575-020-0310-z. 32606456 PMC7447603

[118] Vithayathil M, and Khan SA (2022). Current epidemiology of cholangiocarcinoma in Western countries. J. Hepatol. 77, 1690–1698. 10.1016/j.jhep.2022.07.022. 35977611

[119] Oh D-Y, Ruth He A, Qin S, Chen L-T, Okusaka T, Vogel A, Kim JW, Suksombooncharoen T, Ah Lee M, Kitano M, (2022). Durvalumab plus Gemcitabine and Cisplatin in Advanced Biliary Tract Cancer. NEJM Evid. 1. EVIDoa2200015. 10.1056/EVIDoa2200015. 38319896

[120] Kelley RK, Ueno M, Yoo C, Finn RS, Furuse J, Ren Z, Yau T, Klümpen HJ, Chan SL, Ozaka M, (2023). Pembrolizumab in combination with gemcitabine and cisplatin compared with gemcitabine and cisplatin alone for patients with advanced biliary tract cancer (KEYNOTE-966): a randomised, double-blind, placebo-controlled, phase 3 trial. Lancet 401, 1853–1865. 10.1016/S0140-6736(23)00727-4. 37075781

[121] Kendre G, Murugesan K, Brummer T, Segatto O, Saborowski A, and Vogel A (2023). Charting co-mutation patterns associated with actionable drivers in intrahepatic cholangiocarcinoma. J. Hepatol. 78, 614–626. 10.1016/j.jhep.2022.11.030. 36528236

[122] Nakamura H, Arai Y, Totoki Y, Shirota T, Elzawahry A, Kato M, Hama N, Hosoda F, Urushidate T, Ohashi S, (2015). Genomic spectra of biliary tract cancer. Nat. Genet. 47, 1003–1010. 10.1038/ng.3375. 26258846

[123] Ellis H, Braconi C, Valle JW, and Bardeesy N (2025). Cholangiocarcinoma Targeted Therapies: Mechanisms of Action and Resistance. Am. J. Pathol. 195, 437–452. 10.1016/j.ajpath.2024.11.005. 39730074 PMC11841491

[124] Katoh M, Loriot Y, Brandi G, Tavolari S, Wainberg ZA, and Katoh M (2024). FGFR-targeted therapeutics: clinical activity, mechanisms of resistance and new directions. Nat. Rev. Clin. Oncol. 21, 312–329. 10.1038/s41571-024-00869-z. 38424198

[125] Sia D, Losic B, Moeini A, Cabellos L, Hao K, Revill K, Bonal D, Miltiadous O, Zhang Z, Hoshida Y, (2015). Massive parallel sequencing uncovers actionable FGFR2-PPHLN1 fusion and ARAF mutations in intrahepatic cholangiocarcinoma. Nat. Commun. 6, 6087. 10.1038/ncomms7087. 25608663

[126] Abou-Alfa GK, Sahai V, Hollebecque A, Vaccaro G, Melisi D, Al-Rajabi R, Paulson AS, Borad MJ, Gallinson D, Murphy AG, (2020). Pemigatinib for previously treated, locally advanced or metastatic cholangiocarcinoma: a multicentre, open-label, phase 2 study. Lancet Oncol. 21, 671–684. 10.1016/s1470-2045(20)30109-1. 32203698 PMC8461541

[127] Wu YM, Su F, Kalyana-Sundaram S, Khazanov N, Ateeq B, Cao X, Lonigro RJ, Vats P, Wang R, Lin SF, (2013). Identification of targetable FGFR gene fusions in diverse cancers. Cancer Discov. 3, 636–647. 10.1158/2159-8290.CD-13-0050. 23558953 PMC3694764

[128] Arai Y, Totoki Y, Hosoda F, Shirota T, Hama N, Nakamura H, Ojima H, Furuta K, Shimada K, Okusaka T, (2014). Fibroblast growth factor receptor 2 tyrosine kinase fusions define a unique molecular subtype of cholangiocarcinoma. Hepatology 59, 1427–1434. 10.1002/hep.26890. 24122810

[129] Borad MJ, Champion MD, Egan JB, Liang WS, Fonseca R, Bryce AH, McCullough AE, Barrett MT, Hunt K, Patel MD, (2014). Integrated genomic characterization reveals novel, therapeutically relevant drug targets in FGFR and EGFR pathways in sporadic intrahepatic cholangiocarcinoma. PLoS Genet. 10, e1004135. 10.1371/journal.pgen.1004135. 24550739 PMC3923676

[130] Zingg D, Bhin J, Yemelyanenko J, Kas SM, Rolfs F, Lutz C, Lee JK, Klarenbeek S, Silverman IM, Annunziato S, (2022). Truncated FGFR2 is a clinically actionable oncogene in multiple cancers. Nature 608, 609–617. 10.1038/s41586-022-05066-5. 35948633 PMC9436779

[131] Moeini A, Haber PK, and Sia D (2021). Cell of origin in biliary tract cancers and clinical implications. JHEP Rep. 3, 100226. 10.1016/j.jhepr.2021.100226. 33665585 PMC7902553

[132] Yamamura M, Sato Y, Takahashi K, Sasaki M, and Harada K (2020). The cyclin-dependent kinase pathway involving CDK1 is a potential therapeutic target for cholangiocarcinoma. Oncol. Rep. 43, 306–317. 10.3892/or.2019.7405. 31746435

[133] Zhu Y, Zhang D, Shukla P, Jung YH, Malgulwar PB, Chagani S, Colic M, Benjamin S, Copland JA 3rd, Tan L, (2024). CRISPR screening identifies BET and mTOR inhibitor synergy in cholangiocarcinoma through serine glycine one carbon. JCI Insight 9, e174220. 10.1172/jci.insight.174220. 38060314 PMC10906219

[134] Subbiah V, Lassen U, Élez E, Italiano A, Curigliano G, Javle M, de Braud F, Prager GW, Greil R, Stein A, (2020). Dabrafenib plus trametinib in patients with BRAFV600E-mutated biliary tract cancer (ROAR): a phase 2, open-label, single-arm, multicentre basket trial. Lancet Oncol. 21, 1234–1243. 10.1016/S1470-2045(20)30321-1. 32818466

[135] Guedj N, Blaise L, Cauchy F, Albuquerque M, Soubrane O, and Paradis V (2021). Prognostic value of desmoplastic stroma in intrahepatic cholangiocarcinoma. Mod. Pathol. 34, 408–416. 10.1038/s41379-020-00656-y. 32860001

[136] Thuwajit C, Thuwajit P, Paupairoj A, Chau-In S, Suthiphongchai T, and Thuwajit C (2009). Alpha-smooth muscle actin-positive fibroblasts promote biliary cell proliferation and correlate with poor survival in cholangiocarcinoma. Oncol. Rep. 21, 957–969. 10.3892/or_00000309. 19287994

[137] Bergeat D, Fautrel A, Turlin B, Merdrignac A, Rayar M, Boudjema K, Coulouarn C, and Sulpice L (2016). Impact of stroma LOXL2 overexpression on the prognosis of intrahepatic cholangiocarcinoma. J. Surg. Res. 203, 441–450. 10.1016/j.jss.2016.03.044. 27363654

[138] Affo S, Nair A, Brundu F, Ravichandra A, Bhattacharjee S, Matsuda M, Chin L, Filliol A, Wen W, Song X, (2021). Promotion of cholangiocarcinoma growth by diverse cancer-associated fibroblast subpopulations. Cancer Cell 39, 866–882.e11. 10.1016/j.ccell.2021.03.012. 33930309 PMC8241235

[139] Zhang M, Yang H, Wan L, Wang Z, Wang H, Ge C, Liu Y, Hao Y, Zhang D, Shi G, (2020). Single-cell transcriptomic architecture and intercellular crosstalk of human intrahepatic cholangiocarcinoma. J. Hepatol. 73, 1118–1130. 10.1016/j.jhep.2020.05.039. 32505533

[140] Javle M, Roychowdhury S, Kelley RK, Sadeghi S, Macarulla T, Weiss KH, Waldschmidt DT, Goyal L, Borbath I, El-Khoueiry A, (2021). Infigratinib (BGJ398) in previously treated patients with advanced or metastatic cholangiocarcinoma with FGFR2 fusions or rear-rangements: mature results from a multicentre, open-label, single-arm, phase 2 study. Lancet Gastroenterol. Hepatol. 6, 803–815. 10.1016/S2468-1253(21)00196-5. 34358484

[141] Goyal L, Saha SK, Liu LY, Siravegna G, Leshchiner I, Ahronian LG, Lennerz JK, Vu P, Deshpande V, Kambadakone A, (2017). Polyclonal Secondary FGFR2 Mutations Drive Acquired Resistance to FGFR Inhibition in Patients with FGFR2 Fusion-Positive Cholangiocarcinoma. Cancer Discov. 7, 252–263. 10.1158/2159-8290.CD-16-1000. 28034880 PMC5433349

[142] Goyal L, Meric-Bernstam F, Hollebecque A, Valle JW, Morizane C, Karasic TB, Abrams TA, Furuse J, Kelley RK, Cassier PA, (2023). Futibatinib for FGFR2-Rearranged Intrahepatic Cholangiocarcinoma. N. Engl. J. Med. 388, 228–239. 10.1056/NEJMoa2206834. 36652354

[143] Chaturantabut S, Oliver S, Frederick DT, Kim JJ, Robinson FP, Sinopoli A, Song TY, He Y, Chang YC, Rodriguez DJ, (2025). Identification of potent biparatopic antibodies targeting FGFR2 fusion-driven cholangiocarcinoma. J. Clin. Investig. 135, e182417. 10.1172/JCI182417. 40014401 PMC11996885

[144] Harding JJ, Fan J, Oh DY, Choi HJ, Kim JW, Chang HM, Bao L, Sun HC, Macarulla T, Xie F, (2023). Zanidatamab for HER2-amplified, unresectable, locally advanced or metastatic biliary tract cancer (HERIZON-BTC-01): a multicentre, single-arm, phase 2b study. Lancet Oncol. 24, 772–782. 10.1016/S1470-2045(23)00242-5. 37276871

[145] Bekaii-Saab TS, Yaeger R, Spira AI, Pelster MS, Sabari JK, Hafez N, Barve M, Velastegui K, Yan X, Shetty A, (2023). Adagrasib in Advanced Solid Tumors Harboring a KRAS(G12C) Mutation. J. Clin. Oncol. 41, 4097–4106. 10.1200/JCO.23.00434. 37099736 PMC10852394

[146] Jiang J, Jiang L, Maldonato BJ, Wang Y, Holderfield M, Aronchik I, Winters IP, Salman Z, Blaj C, Menard M, (2024). Translational and Therapeutic Evaluation of RAS-GTP Inhibition by RMC-6236 in RAS-Driven Cancers. Cancer Discov. 14, 994–1017. 10.1158/2159-8290.CD-24-0027. 38593348 PMC11149917

[147] Wu MJ, Shi L, Merritt J, Zhu AX, and Bardeesy N (2022). Biology of IDH mutant cholangiocarcinoma. Hepatology 75, 1322–1337. 10.1002/hep.32424. 35226770

[148] Zhu AX, Macarulla T, Javle MM, Kelley RK, Lubner SJ, Adeva J, Cleary JM, Catenacci DVT, Borad MJ, Bridgewater JA, (2021). Final Overall Survival Efficacy Results of Ivosidenib for Patients With Advanced Cholangiocarcinoma With IDH1 Mutation: The Phase 3 Randomized Clinical ClarIDHy Trial. JAMA Oncol. 7, 1669–1677. 10.1001/jamaoncol.2021.3836. 34554208 PMC8461552

[149] Le DT, Durham JN, Smith KN, Wang H, Bartlett BR, Aulakh LK, Lu S, Kemberling H, Wilt C, Luber BS, (2017). Mismatch repair deficiency predicts response of solid tumors to PD-1 blockade. Science 357, 409–413. 10.1126/science.aan6733. 28596308 PMC5576142

[150] Goeppert B, Roessler S, Renner M, Singer S, Mehrabi A, Vogel MN, Pathil A, Czink E, Köhler B, Springfeld C, (2019). Mismatch repair deficiency is a rare but putative therapeutically relevant finding in non-liver fluke associated cholangiocarcinoma. Br. J. Cancer 120, 109–114. 10.1038/s41416-018-0199-2. 30377340 PMC6325153

[151] Isa T, Tomita S, Nakachi A, Miyazato H, Shimoji H, Kusano T, Muto Y, and Furukawa M (2002). Analysis of microsatellite instability, K-ras gene mutation and p53 protein overexpression in intrahepatic cholangiocarcinoma. Hepatogastroenterology. 49, 604–608. 12063950

[152] Weinberg BA, Xiu J, Lindberg MR, Shields AF, Hwang JJ, Poor man K, Salem ME, Pishvaian MJ, Holcombe RF, Marshall JL, (2019). Molecular profiling of biliary cancers reveals distinct molecular alterations and potential therapeutic targets. J. Gastrointest. Oncol. 10, 652–662. 10.21037/jgo.2018.08.18. 31392046 PMC6657312

[153] Silverman IM, Hollebecque A, Friboulet L, Owens S, Newton RC, Zhen H, Féliz L, Zecchetto C, Melisi D, and Burn TC (2021). Clinicogenomic Analysis of FGFR2-Rearranged Cholangiocarcinoma Identifies Correlates of Response and Mechanisms of Resistance to Pemigatinib. Cancer Discov. 11, 326–339. 10.1158/2159-8290.CD-20-0766. 33218975

[154] Zhang Y, Ma Z, Li C, Wang C, Jiang W, Chang J, Han S, Lu Z, Shao Z, Wang Y, (2022). The genomic landscape of cholangiocarcinoma reveals the disruption of post-transcriptional modifiers. Nat. Commun. 13, 3061. 10.1038/s41467-022-30708-7. 35650238 PMC9160072

[155] Alexandrov LB, Nik-Zainal S, Wedge DC, Aparicio SAJR, Behjati S, Biankin AV, Bignell GR, Bolli N, Borg A, Børresen-Dale AL, (2013). Signatures of mutational processes in human cancer. Nature 500, 415–421. 10.1038/nature12477. 23945592 PMC3776390

[156] Marcus L, Lemery SJ, Keegan P, and Pazdur R (2019). FDA Approval Summary: Pembrolizumab for the Treatment of Microsatellite Instability-High Solid Tumors. Clin. Cancer Res. 25, 3753–3758. 10.1158/1078-0432.CCR-18-4070. 30787022

[157] Farmer H, McCabe N, Lord CJ, Tutt ANJ, Johnson DA, Richardson TB, Santarosa M, Dillon KJ, Hickson I, Knights C, (2005). Targeting the DNA repair defect in BRCA mutant cells as a therapeutic strategy. Nature 434, 917–921. 10.1038/nature03445. 15829967

[158] Vogel A, and Ducreux M; ESMO Guidelines Committee (2025). ESMO Clinical Practice Guideline interim update on the management of biliary tract cancer. ESMO Open 10, 104003. 10.1016/j.esmoop.2024.104003. 39864891 PMC11846563

[159] Job S, Rapoud D, Dos Santos A, Gonzalez P, Desterke C, Pascal G, Elarouci N, Ayadi M, Adam R, Azoulay D, (2020). Identification of Four Immune Subtypes Characterized by Distinct Composition and Functions of Tumor Microenvironment in Intrahepatic Cholangiocarcinoma. Hepatology 72, 965–981. 10.1002/hep.31092. 31875970 PMC7589418

[160] Martin-Serrano MA, Kepecs B, Torres-Martin M, Bramel ER, Haber PK, Merritt E, Rialdi A, Param NJ, Maeda M, Lindblad KE, (2023). Novel microenvironment-based classification of intrahepatic cholangiocarcinoma with therapeutic implications. Gut 72, 736–748. 10.1136/gutjnl-2021-326514. 35584893 PMC10388405

[161] Carapeto F, Bozorgui B, Shroff RT, Chagani S, Solis Soto L, Foo WC, Wistuba I, Meric-Bernstam F, Shalaby A, Javle M, (2021). The Immunogenomic Landscape of Resected Intrahepatic Cholangiocarcinoma. Hepatology 75, 297–308. 10.1002/hep.32150. 34510503 PMC8766948

[162] Arfè A, Fell G, Alexander B, Awad MM, Rodig SJ, Trippa L, and Schoenfeld JD (2020). Meta-Analysis of PD-L1 Expression As a Predictor of Survival After Checkpoint Blockade. JCO Precis. Oncol. 4, 1196–1206. 10.1200/PO.20.00150. 35050777

[163] Kim RD, Chung V, Alese OB, El-Rayes BF, Li D, Al-Toubah TE, Schell MJ, Zhou JM, Mahipal A, Kim BH, (2020). A Phase 2 Multi-institutional Study of Nivolumab for Patients With Advanced Refractory Biliary Tract Cancer. JAMA Oncol. 6, 888–894. 10.1001/jamaoncol.2020.0930. 32352498 PMC7193528

[164] Monge C, Pehrsson EC, Xie C, Duffy AG, Mabry D, Wood BJ, Kleiner DE, Steinberg SM, Figg WD, Redd B, (2022). A Phase II Study of Pembrolizumab in Combination with Capecitabine and Oxaliplatin with Molecular Profiling in Patients with Advanced Biliary Tract Carcinoma. Oncologist 27, e273–e285. 10.1093/oncolo/oyab073. 35274717 PMC8914487

[165] Loeuillard E, Yang J, Buckarma E, Wang J, Liu Y, Conboy C, Pa velko KD, Li Y, O’Brien D, Wang C, (2020). Targeting tumor-associated macrophages and granulocytic myeloid-derived suppressor cells augments PD-1 blockade in cholangiocarcinoma. J. Clin. Investig. 130, 5380–5396. 10.1172/JCI137110. 32663198 PMC7524481

[166] Doki Y, Ueno M, Hsu CH, Oh DY, Park K, Yamamoto N, Ioka T, Hara H, Hayama M, Nii M, (2022). Tolerability and efficacy of durvalumab, either as monotherapy or in combination with tremelimumab, in patients from Asia with advanced biliary tract, esophageal, or head-and-neck cancer. Cancer Med. 11, 2550–2560. 10.1002/cam4.4593. 35611499 PMC9249982

[167] Piha-Paul SA, Oh DY, Ueno M, Malka D, Chung HC, Nagrial A, Kelley RK, Ros W, Italiano A, Nakagawa K, (2020). Efficacy and safety of pembrolizumab for the treatment of advanced biliary cancer: Results from the KEYNOTE-158 and KEYNOTE-028 studies. Int. J. Cancer 147, 2190–2198. 10.1002/ijc.33013. 32359091

[168] Ma L, Hernandez MO, Zhao Y, Mehta M, Tran B, Kelly M, Rae Z, Hernandez JM, Davis JL, Martin SP, (2019). Tumor Cell Biodiversity Drives Microenvironmental Reprogramming in Liver Cancer. Cancer Cell 36, 418–430.e6. 10.1016/j.ccell.2019.08.007. 31588021 PMC6801104

[169] Ma L, Wang L, Khatib SA, Chang CW, Heinrich S, Dominguez DA, Forgues M, Candia J, Hernandez MO, Kelly M, (2021). Single-cell atlas of tumor cell evolution in response to therapy in hepatocellular carcinoma and intrahepatic cholangiocarcinoma. J. Hepatol. 75, 1397–1408. 10.1016/j.jhep.2021.06.028. 34216724 PMC8604764

[170] Zhao X, Guo F, Li Z, Jiang P, Deng X, Tian F, Li X, and Wang S (2016). Aberrant expression of B7-H4 correlates with poor prognosis and suppresses tumor-infiltration of CD8+ T lymphocytes in human cholangiocarcinoma. Oncol. Rep. 36, 419–427. 10.3892/or.2016.4807. 27177355

[171] Xie N, Cai JB, Zhang L, Zhang PF, Shen YH, Yang X, Lu JC, Gao DM, Kang Q, Liu LX, (2017). Upregulation of B7-H4 promotes tumor progression of intrahepatic cholangiocarcinoma. Cell Death Dis. 8, 3205. 10.1038/s41419-017-0015-6. 29235470 PMC5870586

[172] Cheng R, Chen Y, Zhou H, Wang B, Du Q, and Chen Y (2018). B7-H3 expression and its correlation with clinicopathologic features, angiogenesis, and prognosis in intrahepatic cholangiocarcinoma. APMIS 126, 396–402. 10.1111/apm.12837. 29696716

[173] Sibai M, Cervilla S, Grases D, Musulen E, Lazcano R, Mo CK, Davalos V, Fortian A, Bernat A, Romeo M, (2025). The spatial landscape of cancer hallmarks reveals patterns of tumor ecological dynamics and drug sensitivity. Cell Rep. 44, 115229. 10.1016/j.celrep.2024.115229. 39864059

[174] Andersen JB, Spee B, Blechacz BR, Avital I, Komuta M, Barbour A, Conner EA, Gillen MC, Roskams T, Roberts LR, (2012). Genomic and genetic characterization of cholangiocarcinoma identifies therapeutic targets for tyrosine kinase inhibitors. Gastroenterology 142, 1021–1031.e15. 10.1053/j.gastro.2011.12.005. 22178589 PMC3413201

[175] Hayashi A, Misumi K, Shibahara J, Arita J, Sakamoto Y, Hasegawa K, Kokudo N, and Fukayama M (2016). Distinct Clinicopathologic and Genetic Features of 2 Histologic Subtypes of Intrahepatic Cholangiocarcinoma. Am. J. Surg. Pathol. 40, 1021–1030. 10.1097/PAS.0000000000000670. 27259014

[176] Dong L, Lu D, Chen R, Lin Y, Zhu H, Zhang Z, Cai S, Cui P, Song G, Rao D, (2022). Proteogenomic characterization identifies clinically relevant subgroups of intrahepatic cholangiocarcinoma. Cancer Cell 40, 70–87.e15. 10.1016/j.ccell.2021.12.006. 34971568

[177] Bao X, Li Q, Chen J, Chen D, Ye C, Dai X, Wang Y, Li X, Rong X, Cheng F, (2022). Molecular Subgroups of Intrahepatic Cholangiocarcinoma Discovered by Single-Cell RNA Sequencing-Assisted Multiomics Analysis. Cancer Immunol. Res. 10, 811–828. 10.1158/2326-6066.CIR-21-1101. 35604302

[178] Sulpice L, Rayar M, Desille M, Turlin B, Fautrel A, Boucher E, Llamas-Gutierrez F, Meunier B, Boudjema K, Clément B, (2013). Molecular profiling of stroma identifies osteopontin as an independent predictor of poor prognosis in intrahepatic cholangiocarcinoma. Hepatology 58, 1992–2000. 10.1002/hep.26577. 23775819

[179] Jusakul A, Cutcutache I, Yong CH, Lim JQ, Huang MN, Padmanabhan N, Nellore V, Kongpetch S, Ng AWT, Ng LM, (2017). Whole-Genome and Epigenomic Landscapes of Etiologically Distinct Subtypes of Cholangiocarcinoma. Cancer Discov. 7, 1116–1135. 10.1158/2159-8290.CD-17-0368. 28667006 PMC5628134

[180] Hong L, Mei J, Sun X, Wu Y, Dong Z, Jin Y, Gao L, Cheng J, Tian W, Liu C, (2025). Spatial single-cell proteomics landscape decodes the tumor microenvironmental ecosystem of intrahepatic cholangiocarcinoma. Hepatology 83, 57–74. 10.1097/HEP.0000000000001283. 39999448

[181] Chen S, Xie Y, Cai Y, Hu H, He M, Liu L, Liao C, Wang Y, Wang J, Ren X, (2022). Multiomic Analysis Reveals Comprehensive Tumor Heterogeneity and Distinct Immune Subtypes in Multifocal Intrahepatic Cholangiocarcinoma. Clin. Cancer Res. 28, 1896–1910. 10.1158/1078-0432.CCR-21-1157. 34526363

[182] Baretti M, Shekhar S, Sahai V, Shu D, Howe K, Gunchick V, Assarzadegan N, Kartalia E, Zhu Q, Hallab E, (2025). Deep immune profiling of intrahepatic cholangiocarcinoma with CODEX multiplexed imaging. Hepatol. Commun. 9, e0632. 10.1097/HC9.0000000000000632. 39969434 PMC11841852

[183] Wu MJ, Shi L, Dubrot J, Merritt J, Vijay V, Wei TY, Kessler E, Olander KE, Adil R, Pankaj A, (2022). Mutant IDH Inhibits IFNγ-TET2 Signaling to Promote Immunoevasion and Tumor Maintenance in Cholangiocarcinoma. Cancer Discov. 12, 812–835. 10.1158/2159-8290.CD-21-1077. 34848557 PMC8904298

[184] Schram AM, Goto K, Kim DW, Macarulla T, Hollebecque A, O’Reilly EM, Ou SI, Rodon J, Rha SY, Nishino K, (2025). Efficacy of Zenocutuzumab in NRG1 Fusion-Positive Cancer. N. Engl. J. Med. 392, 566–576. 10.1056/NEJMoa2405008. 39908431 PMC11878197

[185] Mertens JC, Fingas CD, Christensen JD, Smoot RL, Bronk SF, Werneburg NW, Gustafson MP, Dietz AB, Roberts LR, Sirica AE, (2013). Therapeutic effects of deleting cancer-associated fibroblasts in cholangiocarcinoma. Cancer Res. 73, 897–907. 10.1158/0008-5472.CAN-12-2130. 23221385 PMC3549008

[186] Amengual J, Gonzalez-Sanchez E, Yáñez-Bartolome M, Sererols-Viñas L, Ravichandra A, Guiton C, Fuste NP, Alay A, Hijazo-Pechero S, Martín-Mur B, (2025). NADPH oxidase 1/4 dual inhibition impairs transforming growth factor-beta protumorigenic effects in cholangiocarcinoma cancer-associated fibroblasts. Signal Transduct. Target. Ther. 10, 257. 10.1038/s41392-025-02347-z. 40820091 PMC12358586

[187] Provenzano PP, Cuevas C, Chang AE, Goel VK, Von Hoff DD, and Hingorani SR (2012). Enzymatic targeting of the stroma ablates physical barriers to treatment of pancreatic ductal adenocarcinoma. Cancer Cell 21, 418–429. 10.1016/j.ccr.2012.01.007. 22439937 PMC3371414

[188] Clift R, Souratha J, Garrovillo SA, Zimmerman S, and Blouw B (2019). Remodeling the Tumor Microenvironment Sensitizes Breast Tumors to Anti-Programmed Death-Ligand 1 Immunotherapy. Cancer Res. 79, 4149–4159. 10.1158/0008-5472.CAN-18-3060. 31248966

[189] Thompson CB, Shepard HM, O’Connor PM, Kadhim S, Jiang P, Osgood RJ, Bookbinder LH, Li X, Sugarman BJ, Connor RJ, (2010). Enzymatic depletion of tumor hyaluronan induces antitumor responses in preclinical animal models. Mol. Cancer Ther. 9, 3052–3064. 10.1158/1535-7163.MCT-10-0470. 20978165

[190] Van Cutsem E, Tempero MA, Sigal D, Oh DY, Fazio N, Macarulla T, Hitre E, Hammel P, Hendifar AE, Bates SE, (2020). Randomized Phase III Trial of Pegvorhyaluronidase Alfa With NabPaclitaxel Plus Gemcitabine for Patients With Hyaluronan-High Metastatic Pancreatic Adenocarcinoma. J. Clin. Oncol. 38, 3185–3194. 10.1200/JCO.20.00590. 32706635 PMC7499614

[191] Huang F, Liu Z, Song Y, Wang G, Shi A, Chen T, Huang S, Lian S, Li K, Tang Y, (2025). Bile acids activate cancer-associated fibroblasts and induce an immunosuppressive microenvironment in cholangiocarcinoma. Cancer Cell 43, 1460–1475.e10. 10.1016/j.ccell.2025.05.017. 40578361

[192] Calderaro J, Seraphin TP, Luedde T, and Simon TG (2022). Artificial intelligence for the prevention and clinical management of hepatocellular carcinoma. J. Hepatol. 76, 1348–1361. 10.1016/j.jhep.2022.01.014. 35589255 PMC9126418

[193] Zeng Q, Klein C, Caruso S, Maille P, Allende DS, Mínguez B, Iavarone M, Ningarhari M, Casadei-Gardini A, Pedica F, (2023). Artificial intelligence-based pathology as a biomarker of sensitivity to atezolizumab-bevacizumab in patients with hepatocellular carcinoma: a multicentre retrospective study. Lancet Oncol. 24, 1411–1422. 10.1016/S1470-2045(23)00468-0. 37951222

[194] Seraphin TP, Mesropian A, Žigutyte_ L, Brooks J, Mauro E, Gris-Oliver A, Pinyol R, Montironi C, Balaseviciute U, Piqué-Gili M, (2025). Artificial intelligence predicts outcome-related molecular profiles and vascular invasion in hepatocellular carcinoma. JHEP Rep. 7, 101592. 10.1016/j.jhepr.2025.101592. 41321933 PMC12657720

[195] Xie J, Pu X, He J, Qiu Y, Lu C, Gao W, Wang X, Lu H, Shi J, Xu Y, (2022). Survival prediction on intrahepatic cholangiocarcinoma with histomorphological analysis on the whole slide images. Comput. Biol. Med. 146, 105520. 10.1016/j.compbiomed.2022.105520. 35537220

[196] Bramel ER, Facciuto M, Torres-Martin M, Park E, Donne R, Sritharan R, Chen L, Ilyas S, Petitjean M, Tocheva A, (2024). Immune checkpoint B7-H4 promotes cholangiocarcinoma growth via TGFb-induced stromal remodelling, unveleing a novel targetable tumor-stroma axis. Hepatology 80, 40–41.

[197] Dapito DH, Mencin A, Gwak GY, Pradere JP, Jang MK, Mederacke I, Caviglia JM, Khiabanian H, Adeyemi A, Bataller R, (2012). Promotion of hepatocellular carcinoma by the intestinal microbiota and TLR4. Cancer Cell 21, 504–516. 10.1016/j.ccr.2012.02.007. 22516259 PMC3332000

[198] Aguirre-Ghiso JA (2021). Translating the Science of Cancer Dormancy to the Clinic. Cancer Res. 81, 4673–4675. 10.1158/0008-5472.CAN-21-1407. 34429327 PMC8562555

[199] Yaniv D, Mattson B, Talbot S, Gleber-Netto FO, and Amit M (2024). Targeting the peripheral neural-tumour microenvironment for cancer therapy. Nat. Rev. Drug Discov. 23, 780–796. 10.1038/s41573-024-01017-z. 39242781 PMC12123372

[200] Malehmir M, Pfister D, Gallage S, Szydlowska M, Inverso D, Kotsiliti E, Leone V, Peiseler M, Surewaard BGJ, Rath D, (2019). Platelet GPIbalpha is a mediator and potential interventional target for NASH and subsequent liver cancer. Nat. Med. 25, 641–655. 10.1038/s41591-019-0379-5. 30936549 PMC12452109

[201] Benson AB, D’angelica MI, Abrams T, Abbott DE, Ahmed A, Anaya DA, Anders R, Are C, Bachini M, Binder D, (2023). NCCN Guidelines^®^ Insights: Biliary Tract Cancers, Version 2.2023. J. Natl. Compr. Canc. Netw. 21, 694–704. 10.6004/jnccn.2023.0035. 37433432

